# Evaluation of deep learning-based feature selection for single-cell RNA sequencing data analysis

**DOI:** 10.1186/s13059-023-03100-x

**Published:** 2023-11-10

**Authors:** Hao Huang, Chunlei Liu, Manoj M. Wagle, Pengyi Yang

**Affiliations:** 1 Computational Systems Biology Unit, Faculty of Medicine and Health, Children’s Medical Research Institute, University of Sydney, Westmead, NSW 2145 Australia; 2 School of Mathematics and Statistics, Faculty of Science, University of Sydney, Camperdown, NSW 2006 Australia; 3 Sydney Precision Data Science Centre, University of Sydney, Camperdown, NSW 2006 Australia; 4 Charles Perkins Centre, University of Sydney, Camperdown, NSW 2006 Australia

## Abstract

**Background:**

Feature selection is an essential task in single-cell RNA-seq (scRNA-seq) data analysis and can be critical for gene dimension reduction and downstream analyses, such as gene marker identification and cell type classification. Most popular methods for feature selection from scRNA-seq data are based on the concept of differential distribution wherein a statistical model is used to detect changes in gene expression among cell types. Recent development of deep learning-based feature selection methods provides an alternative approach compared to traditional differential distribution-based methods in that the importance of a gene is determined by neural networks.

**Results:**

In this work, we explore the utility of various deep learning-based feature selection methods for scRNA-seq data analysis. We sample from Tabula Muris and Tabula Sapiens atlases to create scRNA-seq datasets with a range of data properties and evaluate the performance of traditional and deep learning-based feature selection methods for cell type classification, feature selection reproducibility and diversity, and computational time.

**Conclusions:**

Our study provides a reference for future development and application of deep learning-based feature selection methods for single-cell omics data analyses.

**Supplementary Information:**

The online version contains supplementary material available at 10.1186/s13059-023-03100-x.

## Background

The advances in single-cell RNA sequencing (scRNA-seq) technologies allow the measurement of global gene expression profiles of individual cells, enabling the dissection of heterogeneous cell populations in complex samples that were inaccessible in bulk sequencing data [1]. The large number of genes measured by scRNA-seq technologies results in high-feature-dimensionality and high-feature-redundancy where a large proportion of genes are not informative and therefore may not aid downstream data analyses and interpretation [2]. To this end, various feature selection techniques have been developed and applied for selecting informative genes while filtering those that are uninformative for downstream analyses such as gene marker identification and cell type classification [3]. Among these methods, statistical models that detect the difference in gene expression among cell types are the most frequently applied for feature selection in scRNA-seq data analysis [4, 5]. Compared to these traditional differential distribution-based methods, which are typically referred to as parametric given their reliance on various statistical model assumptions [6], the recent development of deep learning-based feature selection methods alleviates much of model assumptions and thus provides an alternative approach towards dimension reduction and gene selection from scRNA-seq data.

Traditionally, feature selection methods are classified into filters, wrappers, and embedded approaches. Most recently developed deep learning-based feature selection methods belong to the embedded category where various techniques have been proposed for extracting feature importance from neural network models trained on the high-feature-dimensionality data. Based on the feature importance extraction techniques, deep learning-based feature selection methods can be broadly categorized into those based on feature perturbation and those based on gradient back-propagation [7]. In perturbation-based methods, the utility or importance of a feature is determined by perturbing the feature and evaluating its impact on the loss function of the neural networks [8]. Popular methods in this category include local interpretable model-agnostic explanations (LIME) [9] which permutes features of the input samples, FeatureAblation [7], and Occlusion [10] which block each feature or multiple features from the input samples, respectively. In gradient-based methods, this is determined by the change of the gradient from the neural networks [11]. Key examples in this category are layer-wise relevance propagation (LayerRelProp) [12] which determines “weights” of features by sequentially back-propagating gradients across neural network layers, GradientShap [13] which computes the changes of the gradients from the input and a randomly sampled baseline, and deep learning important features (DeepLIFT) [11] which decompose the output from a neural network on features by backpropagating the gradients.

In this work, we applied the above-mentioned six deep learning feature selection methods from the two categories for selecting genes from scRNA-seq data and compared their performance with the differential distribution-based feature selection methods typically used for this task, including DESeq2 [14], Limma-voom [15], scDD [4], and Wilcoxon rank-sum test. In addition, RandomForest [16], an embedded feature selection method, and RelieF [17] a filter method, were included as two other popular methods for feature selection from the machine learning literature. To test these feature selection methods on scRNA-seq datasets with a range of data properties, we leveraged the Tabula Muris [18] and Tabula Sapiens [19] atlases to create datasets with varying numbers of cell types, numbers of cells per cell type, and also ratios of cells from the major and minor cell types. We assessed the performance of each feature selection method on classifying cell types in various scenarios and assessed the impact of different data properties on their performance. We next evaluated the reproducibility of each feature selection method and the concordance across methods. These analyses allow us to identify that genes with diverse expression profiles were selected by deep learning-based methods whereas those selected by DESeq2 and Limma-voom are more similar in their expression profiles. In addition, we benchmarked the computational time used by each feature selection method on datasets with different numbers of cells and number of cell types. These results highlight the extremely fast speed of deep learning-based feature selection methods especially when dealing with large scRNA-seq datasets. Lastly, we evaluated the feature selection methods on a large collection of scRNA-seq datasets generated from human colon tissues for their ability to perform granular cell type and disease status classification across independent samples [20]. Together, this work demonstrates the applicability and utility of deep learning-based feature selection methods for scRNA-seq data analysis and establishes a reference for the future development and application of these methods in single-cell omics studies.

## Results

### Sampling from single-cell atlases to create scRNA-seq datasets for feature selection methods evaluation

**Fig. 1:**
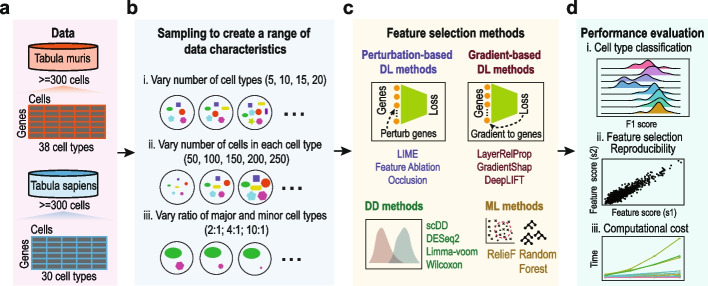
Schematic summaries of the workflow used in this study. **a** Filtering of Tabula Muris and Tabula Sapiens atlases to retain cell types with ≥ 300 cells for subsequent data sampling. **b** Sampling from Tabula Muris and Tabula Sapiens atlases for creating scRNA-seq datasets with varying number of cell types; number of cells in each cell type; and ratios of cells in the major and minor cell types. **c** Deep learning feature selection methods applied for scRNA-seq data analysis in this study were grouped by their category as either perturbation-based and gradient-based methods. Popular differential distribution-based and machine-learning-based methods were included for comparison. **d** Genes selected by each feature selection method were evaluated for their utility in cell type classification. Reproducibility of feature selection results and computational time were also assessed for each feature selection method

To evaluate feature selection methods on scRNA-seq data analysis and assess the impacts of various data characteristics on their performance, we leveraged the Tabula Muris and Tabula Sapiens single-cell atlases to create scRNA-seq datasets with a range of properties with the aim of dissecting their effects on the performance of each feature selection method. The evaluation workflow is summarized in Fig. 1. In particular, we randomly sampled from the two single-cell atlases (Fig. 1a) to create datasets with (i) varying numbers of cell types from 5, 10, 15, to 20 with each containing 200 cells; (ii) varying numbers of cells in each cell type from 50, 100, 150, 200, to 250 with the numbers of cell types set as 10 or 20; and (iii) varying the ratios of cells from the major and minor cell types from 2:1, 4:1, to 10:1 with the numbers of cell types set as 10 or 20 and half as major and the other half as minor cell types (Fig. 1b) (see the “Methods” section for details).

Among the feature selection methods (Fig. 1c), we implemented three popular perturbation-based methods including LIME, FeatureAblation, and Occlusion, and also three popular gradient-based methods including LayerRelProp, GradientShap, and DeepLIFT. In particular, we used a multilayer perceptron neural network for these methods (see the “Methods” section for details). For comparison to deep learning-based feature selection methods, differential distribution-based methods of DESeq2, Limma-voom, scDD, and Wilcoxon rank-sum test and traditional machine learning-based methods including RandomForest, an embedded feature selection method, and RelieF, a filter method for feature selection, were included in the evaluation. To assess the performance of feature selection methods, we computed (i) the utility of top genes selected by each method (top 5, 10, and 20 genes) on classifying cell types using a K-nearest neighbor (KNN) classifier and a Support Vector Machine (SVM) classifier and recorded their sensitivity, specificity, and F1 score; (ii) reproducibility of gene ranks based on the correlation of their importance scores (e.g., weight, statistics, see Methods for details) by subsampling of datasets; and (iii) computational time used by each method with respect to increasing number of cells and cell types in the datasets. The above-created data characteristics and evaluation metrics provide a multifaceted framework for assessing the performance of feature selection methods for scRNA-seq data analysis.

### Performance of feature selection methods on cell type classification and impact of number of cell types

**Fig. 2:**
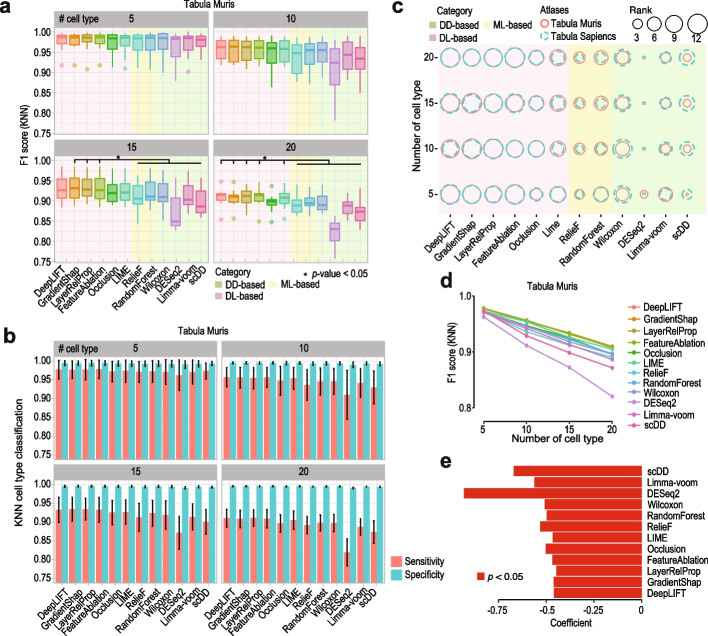
Performance of feature selection methods and impact of cell type numbers on gene selection for classifying cell type using Tabula Muris atlas. **a** F1 scores calculated from a *k*-nearest neighbor classifier (KNN; *k* = 7) using the union of top-10 cell type marker genes selected by each feature selection method for classifying cell types. Higher F1 scores indicate more accurate cell type classification. The number of cell types varies from 5, 10, 15 to 20 in the datasets and cell type classification was repeated 10 times, by random sampling from Tabula Muris altas, for each feature selection method in each setting to capture the classification variability. Statistical significance (* *p* < 0.05 based on Wilcox rank sum tests) was denoted if every deep learning-based method outperformed every traditional method. **b** Similar to **a** but quantifying KNN cell type classification performance by sensitivity and specificity. **c** For numbers of cell types set at 5, 10, 15, and 20, balloon plots summarizing the ranks of median F1 scores from KNN on datasets sampled from Tabula Muris and Tabula Sapiens atlases. The size of the balloon represents the rank of the method, the larger the better its performance. **d** mean F1 scores from **a** plotted against the increasing number of cell types, and **e** coefficients of slopes from least squares fitted lines to F1 scores in **d**

We first evaluated the utility of the genes selected by each feature selection method for cell type classification by varying the number of cell types in the datasets. As expected, the overall F1 scores of both KNN and SVM classifiers reduced when the number of cell types increased in the datasets sampled from Tabula Muris atlas presumably due to the increasing difficulty in the classification tasks (Fig. 2a and Additional file 1: Fig. S1a). Notably, the difference in performance among deep learning-based feature selection methods and traditional methods is more apparent with deep learning-based methods performing better when the datasets contain larger numbers of cell types (e.g., 15 and 20) and hence more difficult to classify. In particular, we found that in most cases DeepLIFT, GradientShap, LayerRelProp, and FeatureAblation have higher F1 scores compared to other methods. Further breakdown of the classification performance of selected features by each method into sensitivity and specificity reveals that features selected from most methods led to generally high specificity whereas the sensitivity varies more among methods and dropped considerably with the increasing number of cell types in the datasets (Fig. 2b and Additional file 1: Fig. S1b). The high specificity is likely due to the use of the “one versus all” approach for quantifying classification performance (see the “Methods” section) where the precision is high when a cell is classified to a cell type, yet discriminating all cells of a cell type from the rest of the cell types is challenging, leading to varying sensitivity and F1 scores that distinguish the performance of each method. Similar results were observed from datasets sampled from Tabula Sapiens atlas (Additional file 1: Fig. S2a, b) and the ranks of methods by their median F1 scores further demonstrate the high concordance of results from the two single-cell atlases for most methods except scDD, which performed better for data sampled from Tabula Sapiens atlas, and to a lesser degree LIME, which performed better for data sampled from Tabula Muris atlas (Fig. 2c). While the above results were from using the union of top-10 genes selected from each cell type in a dataset, we also varied the number of top genes used to top-5 and top-20 (Additional file 1: Fig. S3a, b). Again, we found similar results from using both KNN and SVM classifiers on both atlases suggesting that the three gradient-based deep learning feature selection methods, DeepLIFT, GradientShap, and LayerRelProp, and FeatureAblation, a perturbation-based deep learning feature selection method, are among the best-performing ones in feature selection from scRNA-seq data for cell type classification.

To further investigate the decrease in cell type classification accuracy for each feature selection method when the number of cell types increases in the datasets, we fitted a least squares line to the F1 scores across the number of cell types for each method (Fig. 2d) and extracted the coefficient of the slope (Fig. 2e). The analysis of the coefficients suggests that all feature selection methods show a significant reduction in classification accuracy against the increasing number of cell types in the datasets. Notably, the three differential distribution-based methods (i.e., scDD, Limma-voom, DESeq2) are more affected by the increasing number of cell types in the datasets with steeper decrease in F1 scores in cell type classification compared to deep learning-based methods. Together, these results demonstrate the high performance of a subset of deep learning-based feature selection methods (i.e., DeepLIFT, GradientShap, LayerRelProp, FeatureAblation) for cell type classification, especially when dealing with datasets with large numbers of cell types.

### The effect of number of cells on feature selection methods

**Fig. 3:**
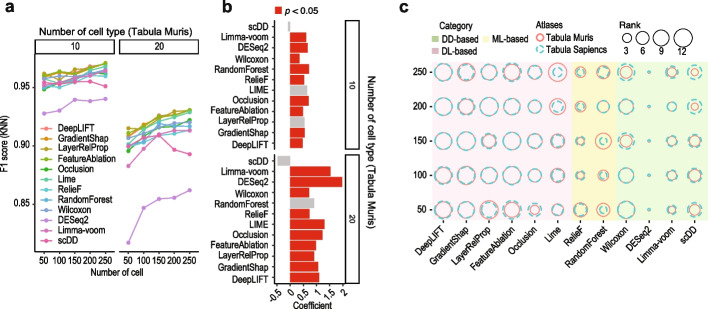
Impact of number of cells on cell type classification using the union of top-10 cell type marker genes selected by each feature selection method. **a** Mean F1 scores of KNN classification of 10 or 20 cell types (sampled from Tabula Muris atlas) each with number of cells set as 50, 100, 150, 200, and 250. **b** Coefficients of slopes from least squares fitted lines to F1 scores in **a** across the number of cells. **c** For numbers of cells set as 50, 100, 150, 200, and 250 and number of cell types fixed at 20, balloon plots summarizing the ranks of median F1 scores from KNN on datasets sampled from Tabula Muris and Tabula Sapiens atlases. The size of the balloon represents the rank of the method, the larger the better its performance

Besides the change in the number of cell types, the number of cells can change dramatically in scRNA-seq datasets depending on the sample size and the depth of profiling. To this end, we evaluated the impact of the number of cells on gene selection results for cell type classification by varying the number of cells from 50 to 250 while holding the number of cell types at 10 and 20, respectively. For most feature selection methods, we found that the presence of more cells of each cell type in the datasets led to a clear increase in F1 scores for both data sampled from Tabula Muris (Fig. 3a) and Tabula Sapiens (Additional file 1: Fig. S4a) atlases. The coefficient analysis of the least squares fitted line to the F1 scores suggests that increasing the number of cells in the datasets has a greater impact on the F1 scores when the number of cell types is larger (i.e., 20 versus 10) (Fig. 3b and Additional file 1: Fig. S4b).

While the above analyses reveal the impact of the number of cells on feature selection methods, the ranking of their performance in terms of F1 scores demonstrates that deep learning-based methods in most cases outperform traditional methods, and the results are largely independent of the classification models and the number of cells included in the datasets (Fig. 3c and Additional file 1: Fig. S4c). Further dissection of the classification performance to sensitivity and specificity suggests again that specificity of cell type classification is high across all methods and data settings and sensitivity of cell type classification is the main driver in the difference of performance among methods (Additional file 1: Fig. S5a, b). These results together unveil the effect of the number of cells on feature selection methods and highlight the robustness of superior performance we observed from deep learning-based methods over traditional methods.

### Imbalance ratio of number of cells from major and minor cell types affects the performance of feature selection methods

**Fig. 4:**
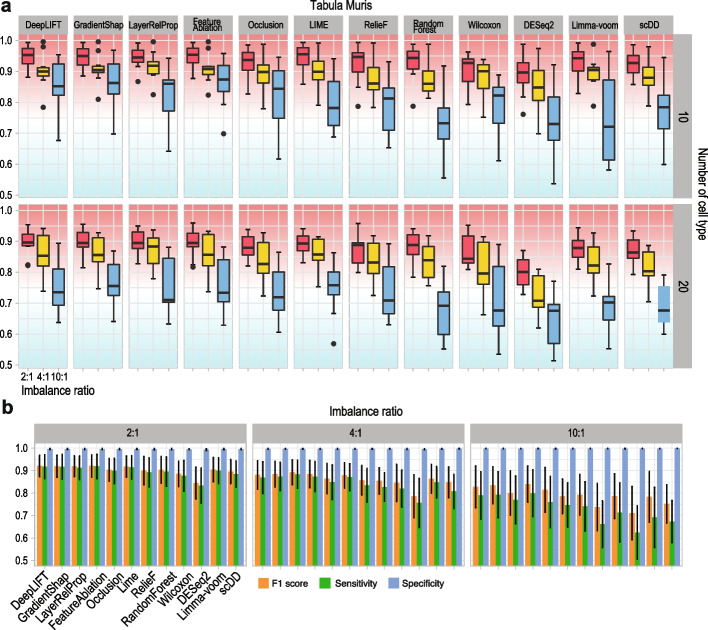
Impact of the number of imbalance ratios on selected genes for cell type classification. **a** F1 scores from KNN classification of minor cell types on datasets with imbalance ratios of number of cells from major and minor cell type set as 2:1, 4:1, and 10:1, and numbers of cell types set as 10 and 20. Each setting was repeated 10 times by random sampling from Tabula Muris atlas for evaluating variability in performance. **b** Performance breakdown of **a** to sensitivity and specificity. F1 scores are included for reference

Since it is common for a scRNA-seq dataset to contain both major and minor cell types, we set out to evaluate the impact of imbalance ratios of number of cells from major and minor cell types on the performance of feature selection methods. By subsampling from Tabula Muris and Tabula Sapiens atlases, we created datasets with major and minor cell types with imbalance ratios of 2:1, 4:1, and 10:1 (see the “Methods” section for details). As expected, F1 scores from using the union of top-10 genes selected from each cell type show a clear reduction in performance on KNN classification of minor cell types when the imbalance ratio increases using both data sampled from Tabula Muris (Fig. 4a) and Tabula Sapiens (Additional file 1: Fig. S6) atlases. Presumably, this is due to increased difficulties in classifying minor cell types when the imbalance ratio increases. Further breakdown of the cell type classification performance visualizes the similar trend of decreasing sensitivity and F1 score across the increasing imbalance ratios while the specificity remains high for all methods (Fig. 4b). We also assessed the performance of KNN and SVM by varying the number of top-ranked genes used for classifying cells from 5 to 10 and then to 20 (Additional file 1: Fig. S7a, b). These assessments further confirm that DeepLIFT, GradientShap, LayerRelProp, and FeatureAblation are among the top-performing feature selection methods across most datasets and support that the ranking of the classification results from each feature selection methods is largely invariant in terms of the numbers of features used, data sources, and the imbalance ratios.

### Feature selection reproducibility within and across feature selection methods

**Fig. 5:**
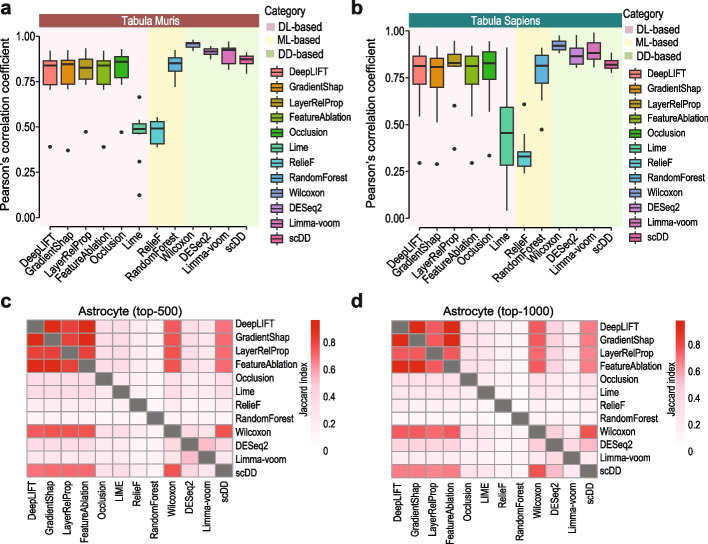
Reproducibility of gene selection results. Reproducibility of gene selection results from each feature selection method on repeatedly sampled datasets (see the “Methods” section) from **a** Tabula Muris and **b** Tabula Sapiens are quantified using Pearson’s correlation coefficient. **c**,** d** Pairwise overlaps of top-500 and top-1000 genes selected by each method for Astrocyte as quantified by Jaccard index

Feature selection reproducibility is another critical consideration especially in scRNA-seq data analysis where the selected genes are frequently interpreted as cell type markers. To evaluate the reproducibility within each feature selection method, we sampled from the two atlases 10 cell types and repeatedly sampled 200 cells for each cell type 10 times. The pairwise Pearson’s correlations of the statistics reported from each feature selection method on the 10 repetitions were used to quantify the reproducibility of the method. As expected, differential distribution-based methods show very high reproducibility in their reported feature statistics in both atlases (Fig. 5a, b). In comparison, we found that, except LIME, the other five deep learning-based feature selection methods also had relatively high reproducibility in their feature importance scores, albeit lower than DESeq2 and Limma-voom (Fig. 5a, b). Notably, LIME and RelieF had relatively low reproducibility in their feature selection results. The low reproducibility of RelieF was reported previously and is probably due to the nearest neighbor selection procedure employed by the algorithm [21].

We next wondered if similar sets of genes were selected by different feature selection methods, that is, if the selected genes are reproducible across different feature selection methods. To answer this question, we quantified the overlaps of the top-ranked 500 and 1000 genes from each pair of methods and in different cell types (Fig. 5c, d and Additional file 1: Fig. S8). We found that DeepLIFT, GradientShap, LayerRelProp, and FeatureAblation selected genes show very high overlaps whereas the genes selected by other methods had only moderate overlaps. Interestingly, genes selected by Wilcoxon rank-sum test and scDD overlapped quite well with the four other deep learning-based methods but can be variable in different cell types (Additional file 1: Fig. S8). Given that large proportions of top-ranked genes from deep learning-based methods and those from DESeq2 and limma-voom are different, we investigated the expression profiles of the top-ranked genes from these methods. As representative examples, we found that genes selected by deep learning-based methods such as DeepLIFT (Additional file 1: Fig. S9a) and Occlusion (Additional file 1: Fig. S9b) show both specificity to their respective cell type and diversity in expression patterns. Whereas the top-ranked genes from DESeq2 (Additional file 1: Fig. S9c) and Limma-voom (Additional file 1: Fig. S9d) show only specificity to the cell type but very little diversity in their expression patterns. Together, these results demonstrate the high within and across method reproducibility of a subset of deep learning-based feature selection methods including DeepLIFT, GradientShap, LayerRelProp, and FeatureAblation.

### Computational time of each feature selection method

**Fig. 6:**
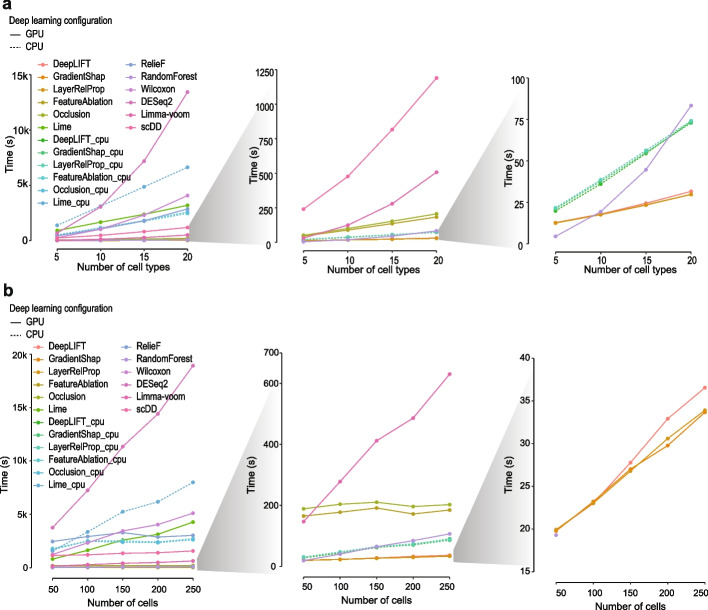
Computational time of feature selection methods. **a** Running time of each feature selection method on datasets sampled from Tabula Muris with the number of cell types increases from 5 to 20 with an increment of 5 and the number of cells in each cell type held as 200. **b** Running time of each feature selection method on datasets with the number of cells increases from 50 to 250 with an increment of 50 and the number of cell types held as 20. Deep learning-based methods were applied with GPU and CPU configurations, respectively

We benchmarked the computational time used by each feature selection method in the settings (i) varying numbers of cell types and (ii) varying numbers of cells (Fig. 6 and Additional file 1: Fig. S10). Among all methods, DESeq2 was the slowest in most cases and scaled poorly especially with the number of cell types (Fig. 6a). Wilcoxon rank-sum test was also one of the slowest methods especially on datasets with a large number of cells and cell types. Like DESeq2, limma-voom uses only 1 CPU core at a time, and therefore both methods scale linearly with respect to the number of cells. Nevertheless, it was much faster than DESeq2 and scDD which uses multiple CPU cores (24 cores were used in this study). For the two machine learning-based methods, while RandomForest is one of the fastest methods, RelieF appears to be significantly slower in comparison. Compared to traditional methods, except LIME, other deep learning-based feature selection methods are extremely fast when applied using GPUs. Specifically, perturbation-based methods scaled well with respect to the number of cells in the datasets, and mostly notably, gradient-based methods are orders of magnitude faster than other methods, ranking features for each of all cell types in less than 40 s for datasets containing 20 cell types and 5000 cells (Fig. 6a, b). When applied to CPUs, gradient-based methods remain fast while perturbation-based methods become significantly slower. These results demonstrate the significant advantage in terms of computational time for deep learning-based feature selection methods over traditional methods especially when dealing with large scRNA-seq datasets with many cell types and large number of cells in each cell type.

### Characteristics of selected genes and further evaluation on scRNA-seq data with granular cell types and across samples

We next explored the characteristics of the genes selected by different feature selection methods. We found that genes selected by deep learning-based feature selection methods show relatively low to moderate expression levels especially when compared to scDD and to a lesser degree RelieF (Additional file 1: Fig. S11a), and have comparable numbers of overlaps with a list of variably expressed transcription factors (VETFs) as defined in [22] for their utility in marking cell types (Additional file 1: Fig. S11b). Lastly, we also evaluated the feature selection methods on another large collection of scRNA-seq data generated from profiling human tissues from healthy individuals and ulcerative colitis patients, and with cell type grouped into three main categories (i.e., stromal, immune, and epithelial cells) each with more granular cell type annotations. We first evaluated the performance of feature selection methods on discriminating granular cell types using samples from healthy individuals. Given that these samples were obtained from multiple individuals, we used a subset of them for feature selection and the rest were used for evaluation (see the “Methods” section), allowing the utility of selected genes to be assessed across independent samples. Consistent with the results from Tabula Muris and Tabula Sapiens atlases, we found that in most cases, deep learning-based feature selection methods led to better cell type classification across independent samples compared to traditional methods (Additional file 1: Fig. S12a). We also tested the utility of selected genes for classifying healthy samples and those from ulcerative colitis patients (Additional file 1: Fig. S12b). While the performance of genes selected by different methods is similar to each other, our results demonstrate that these selected genes indeed can also discriminate samples based on their disease status. Together, these results provide additional views of selected genes and their utility in scRNA-seq data analysis.

## Discussion

Feature selection methods have been demonstrated to be effective in analyzing scRNA-seq data [3] such as improving cell type classification (Additional file 1: Fig. S13). Most traditional differential distribution-based methods are known as filter methods in feature selection literature and calculate statistics for each gene individually and for each cell type sequentially [3]. In comparison, the deep learning-based feature selection methods employed in this study are classified as embedded methods and evaluate groups of genes for all cell types at the same time using the neural network models. The computational speed advantage of ranking all genes for all cell types simultaneously is clearly seen in Fig. 6 and Additional file 1: Fig. S10. The other advantage of such embedded approaches using deep learning-based feature selection methods is the lower redundancy of select features as genes with diverse expression profiles that together lead to better cell type separation can be captured by the model. This is reflected as the diversity of the expression profiles of the selected genes (Additional file 1: Fig. S9). The inclusion of genes with low diversity in expression patterns, as those selected by DESeq2 and Limma-voom (Additional file 1: Fig. S9), may introduce high redundancy and therefore may not facilitate additional gain in cell type discrimination. These findings argue for the application of deep learning-based feature selection methods especially when dealing with large-scale scRNA-seq data and the selection of a diverse panel of genes for accurate cell type classification is a key aim.

Deep learning-based feature selection by embedded approaches relies on various methods to extract feature importance from a neural network model that intrinsically learns the informativeness of genes in scRNA-seq data. This may contribute to the high overlap of selected genes among deep learning-based methods implemented in this study (Fig. 5 and Additional file 1: Fig. S8). While wrapper methods [3] can also be employed with deep learning models for feature selection, these approaches are much less scalable in dealing with high-feature-dimensionality and therefore may not be suited for gene selection from high-dimensional scRNA-seq data. It is also useful to note that both the deep learning-based feature selection methods and other alternative methods (e.g., differential distribution-based methods) included in this work rely on cell type labels. The recent development of unsupervised deep learning-based feature selection techniques (e.g., [23, 24]) provides an alternative approach when such information is not available.

We found that the cell type classification accuracy in terms of F1 score (Fig. 4a) and sensitivity (Fig. 4b) on minor cell types decreased significantly under high imbalance ratio settings (i.e., 10:1). The drop in performance was most prominent for traditional differential distribution-based methods albeit significant decrease was also observed for deep learning-based methods. These findings highlight the need for methods that could account for and/or alleviate the imbalance of number of cells among major and minor cell types [25]. We also found a slightly lower feature selection reproducibility for most deep learning-based methods (except LIME, which has much lower feature selection reproducibility) compared to DESeq2 and Limma-voom (Fig. 5a, b). This is not unexpected given the significantly higher flexibility and hence variability in the deep learning models compared to traditional statistical models. One potential approach for improving reproducibility in deep learning models is to perform ensemble learning with deep learning models [26], in which the increased model stability from ensemble learning can produce more reproducible feature ranking. Future research is required to design and assess the performance of such ensemble deep learning feature selection methods.

Given the underlying hypothesis testing framework used by most differential distribution-based methods, one advantage of these methods is their ability to directly estimate uncertainties of selected genes for controlling false discovery rate (FDR). Nevertheless, deep learning-based feature selection methods can also provide FDR estimation using permutation approaches such as computing importance scores of genes from datasets with the original cell type annotation perturbed randomly. This is a typical procedure also used by various differential distribution-based method especially when the data distribution does not fit the model assumption (e.g., normally distributed).

Finally, most traditional differential distribution-based methods (except Wilcoxon rank-sum test included in the evaluation) are referred to as parametric models in that they require the data distribution to largely fit the assumption made by the statistical model [27]. Compared to traditional parametric models, deep learning-based methods are “non-parametric” and do not make specific assumptions on the data distribution. Hence, deep learning-based feature selection methods are more flexible to accommodate data with different distributions and could be applied to selecting features from other data modalities generated from various single-cell omics technologies, such as scATAC-seq data [28] and single-cell multimodal omics data [29], with minimum changes in these models. We therefore anticipate further development and increasing application of deep learning-based feature selection methods to other types of single-cell omics data.

## Conclusions

Feature selection is an essential technique for scRNA-seq data analysis, especially for downstream biomarker identification and cell type classification. Recent developments in deep learning methods have provided new approaches for feature selection. Yet, the utility of deep learning-based feature selection techniques for scRNA-seq data analysis has not been systematically evaluated. In this study, we have performed a comprehensive evaluation study on six deep learning-based feature selection methods for scRNA-seq data analysis, including tasks such as cell type classification, feature selection reproducibility and diversity, and computational efficiency using datasets with various characteristics. Compared to traditional feature selection methods, we found that deep learning-based feature selection methods are suitable for large-scale scRNA-seq data, given their abilities to simultaneously evaluate and select a panel of genes for cell type classification, which give them the advantages compared to the methods that evaluate genes individually and separate from tasks such as cell type classification. Our results highlight the utility of deep learning-based feature selection methods for scRNA-seq data and identify possible direction for their future development.

## Methods

### Preprocessing of Tabula Muris and Tabula Sapiens atlases

Tabula Muris and Tabula Sapiens atlases contain high-coverage of various cell types from mouse and human, respectively, and high-quality annotation of cells to their respective cell types [18, 19] and were used for creating datasets with cell type ground truth and pre-defined characteristics in this study. Specifically, data generated by FACS sorting and Smart-seq2 protocol were obtained from the atlases’ website. For Tabula Muris atlas, it contains 53,760 cells from 81 cell types and 20 organs of 7 mice. For Tabula Sapiens, the atlas contains 58,870 cells from 133 cell types and 24 organs of 13 donors. A preprocessing step was applied to the two atlases. This includes removing cells that have zero expression of all genes and genes that have zero expression in all cells followed by excluding cell types that have less than 300 cells. This resulted in a count matrix of 23,043 genes and 39,712 cells with 38 cell types from Tabula Muris atlas and a count matrix of 56,119 genes and 27,051 cells with 30 cell types from the Tabula Sapiens atlas.

### Sampling from Tabula Muris and Tabula Sapiens atlases

We performed various random sampling procedures to create datasets with cell type ground truth and pre-defined characteristics. First, to test the impact of the number of cell types on the feature selection methods, for each atlas, we randomly selected from all cell types 5 to 20 cell types passed the preprocessing (with an increment of 5) and for each selected cell type we randomly sampled 200 cells. This procedure was repeated 10 times with random seedings to evaluate the variability in performance and together resulted in a total of 80 datasets.

Second, to evaluate the impact of the number of cells on feature selection methods, we created datasets with the number of cell types fixed at 10 or 20 by randomly selecting from all available cell types for each atlas. For each selected cell type, we next randomly sampled the cells to create varying numbers of cells from 50 to 250 with an increment of 50. This process was repeated 10 times with random seedings and resulted in 200 datasets.

Lastly, we tested the effect of imbalance ratios of cells between major and minor cell types. Similar to the above settings, for each atlas, we started by randomly selecting 10 or 20 cell types from all cell types passed the preprocessing. For these selected cell types, half were treated as major cell types and the other half as minor cell types, where we randomly sampled 200 cells for each major cell type. Next, different imbalance ratios of cells between major and minor were created by randomly sampling 100 (2:1), 50 (4:1), and 20 (10:1) cells for each of the minor cell types. Again, this procedure was repeated 10 times with random seedings and resulted in 120 datasets.

The count data of each above-created dataset was then normalized into log count data using the “LogNormCount” function from R package scater [30]. Filtering of lowly expressed genes were then applied to each dataset before performing feature selection. Specifically, for each dataset, we filtered out genes that are expressed in less than 1% of cells in each of all cell types.

### Reproducibility assessment by random sampling of cells

To assess the reproducibility of feature selection results for each method, for each atlas, we first randomly selected 10 cell types from all cell types and then randomly sampled 200 cells for each cell type. This process was repeated 10 times with different random seedings for sampling cells while keeping the same selected 10 cell types. This resulted in 10 datasets containing the same cell types, but different sets of cells sampled from each atlas. To ensure the feature dimensions are consistent across the 10 sampled datasets for each atlas, only log count normalization was performed whereas the filtering step for genes was excluded.

### Colon tissue profiling scRNA-seq data collection

A collection of scRNA-seq data profiling colon tissues from healthy individuals and ulcerative colitis patients [20] with granular cell type annotation from the three cell type categories, stromal, immune, and epithelial cells, was included for evaluation. Cells that have zero expression of all genes and genes that are expressed in less than 1% of cells in each of all cell types were filtered. The count data of each dataset was then normalized into log count data using the “LogNormCount” function from R package scater [30]. This resulted in three count matrices of 17,486 genes × 8219 cells with 10 cell types, 18,717 genes × 49,973 cells with 14 cell types, and 18,253 genes × 49,664 cells with 12 cell types, respectively.

### Deep learning-based feature selection methods

Three gradient-based feature selection methods, LayerRelProp, DeepLIFT, and GradientShap, and three perturbation-based feature selection methods, Occlusion, FeatureAblation, and LIME, are included in this study.

#### LayerRelProp

LayerRelProp uses back-propagation to recursively propagate the importance scores from the output layer to the input layer [12]. LayerRelProp follows the conservation of total relevance in each layer as follows:

$$f\left(x\right)=\dots =\sum_{i}{R}_{i}^{l+1}=\sum_{i}{R}_{i}^{l}=\dots =\sum_{i}{R}_{i}^{1},$$

where ${R}_{i}^{l}$ is the importance score for neuron $i$ at the ${l}^{th}$ layer, $f$ is the classifier, $x$ is the input, and each importance score is defined as the sum of incoming messages:

$${R}_{i}^{l}={\sum }_{k}{R}_{i\leftarrow k}^{l,l+1}$$

where $k$ is the neuron in the ${(l+1)}^{th}$ layer and $i$ is the input for neuron $k$. As LayerRelProp performs back-propagation recursively from one layer to its previous layer, it generates an importance score for each input feature.

#### DeepLIFT

DeepLIFT decomposes the output prediction of a neural network on a specific input by back-propagating the contributions of all neurons to each input feature [11]. First, given a reference input ${x}^{0}$ with the network score ${t}^{0}$ and an input vector $x$ with network output score $t$, DeepLIFT defines their difference as:

$$\Delta x=x- {x}^{0},$$

$$\Delta t=t- {t}^{0},$$

Then, DeepLIFT assigns the importance scores to each layer according to the following summation-to-delta:

$${\sum }_{i}{R}_{\Delta {x}_{i}\Delta t}=\Delta t,$$

Where ${x}_{i}$ represents the neurons that are necessary and sufficient to compute $t, {R}_{\Delta {x}_{i}\Delta t}$ represents the amount of difference-from-reference in $t$ that is attributed to the difference-from-reference of ${x}_{i}$. In order to compute the importance scores, DeepLIFT defines a multiplier based on a chain rule:

$$m_{\Delta x\Delta t}=\frac{R_{\Delta x\Delta t}}{\Delta x},m_{\Delta x_{\mathrm i}\Delta t}=\sum\nolimits_jm_{\Delta x_i\Delta z_j}m_{\Delta z_i\Delta t},$$

where ${m}_{\Delta x\Delta t}$ represents the contribution of $\Delta x$ to $\Delta t$ divided by $\Delta x$ and ${m}_{\Delta {x}_{i}\Delta t}$ is the quantity computed by the chain rule.

#### GradientShap

GradientShap leverages “Shapley” values from cooperative game theory with a gradient approach to calculate feature importance score [13]. First, Gaussian noise was added to each input sample $k$ times to generate $k$ examples:

$${x}_{i,j}={x}_{i}+{n}_{j},$$

where ${x}_{i}$ represents the ${i}^{th}$ sample, ${n}_{j}$ is ${j}^{th}$ noise and $j\in \{\mathrm{1,2},\dots ,k\}$, $n\sim N\left(\mathrm{0,1}\right)$. In this work, we use the default value of $k=5$.

Then for each input, GradientShap generates a random baseline and chooses a random point along the path between the random baseline and the input to compute the gradient of outputs with respect to the random point.

$${\Delta }_{i,j}= \frac{\partial t}{\partial (\alpha ({x}_{i,j}-{x}_{0}))},$$

Where ${x}^{0}$ is the random baseline, $t$ is the output*,* and $\alpha \in (\mathrm{0,1})$ decides the location of the random point. The importance scores for the ${i}^{th}$ sample are calculated as:

$${R}_{i}=\frac{1}{k}{\sum }_{j}{(\Delta }_{i,j}{(x}_{i,j}-{x}_{0}))$$

and the final importance scores for features are the sum of the importance scores across all samples.

#### Occlusion

Occlusion is a perturbation-based approach for computing feature importance and involves replacing a contiguous sliding region of the input layer of the neural network with some baseline values and comparing the difference with the output of the original model [10]. In our implementation, we define the size of the sliding region of the input layer as three and the baseline value for the sliding region as 0.

#### FeatureAblation

Instead of defining a sliding region of the input layer as in Occlusion, FeatureAblation computes the feature importance by replacing each input feature with a given baseline and computing the difference in the output [7]. We leveraged the default settings that each scalar value within each input tensor is taken as a feature and replaced independently, and the baseline value of 0 was used.

#### LIME

LIME is a model-agnostic approach and can be applied to various machine learning models [9]. Similar to other perturbation-based methods, LIME first perturbates a single sample by randomly masking the feature values, where each feature under evaluation is set to the original input while others are set to 0. Then, LIME trains an interpretable surrogate model (e.g., linear model) by sampling points with the feature under evaluation around a specified input example. In our implementation, the number of sampling points was set to 500. The coefficients of the trained surrogate model represent the importance of the feature under evaluation.

### Setup of deep learning-based feature selection workflow

To enable comparison and minimize the network architecture-specific effect on the above deep learning-based feature selection methods, the same neural network model was used for all deep learning-based feature selection methods. Specifically, the model consists of three fully connected layers, with the input dimension as the number of genes from the input datasets and the output dimension as the number of cell types. The numbers of neurons in the two hidden layers were set to be 1024 and 512, and the activation functions set as LeakyReLU [31] and ReLU [32] respectively. For LayerRelProp, both activation functions were set as ReLU [32] as it has not yet supported LeakyReLU [31]. The output of the neural network model is a probability vector of cell type prediction through SoftMax activation function. During the training process, we used cross-entropy loss for the neural network with a batch size and epochs set as 64 and 20, respectively. After the neural networks were trained on classifying cell types in the training scRNA-seq data, we applied the above six feature selection techniques for each cell type, generating importance scores for each gene within every cell. Next, by aggregating the importance scores across all cells associated with a specific cell type, we obtained the overall importance score for each gene within a particular cell type. Subsequently, the genes were ranked based on their cumulative importance scores, giving higher ranks to those that better distinguish and characterize their respective cell types. This workflow allows us to identify the critical genes that play a pivotal role in the classification of distinct cell types and capture the underlying biological variation.

### Differential distribution-based and machine learning-based feature selection methods

For differential distribution-based methods, two most popular differential expression methods, DESeq2 [14] and Limma-voom [15], a recent method that is specifically designed for scRNA-seq data, scDD [4], and the Wilcoxon rank-sum test were included for comparison in this study. To select genes specific to each cell type, the expression of a gene in a cell type was compared to its expression in other cell types. For DESeq2 and Limma-voom, genes were ranked and selected by “stats” and “t” statistics, respectively. For scDD, only *p*-values were reported from the package and we used the -log10 of the *p*-value and the sign of the log2 fold change of the gene expression to rank and select genes. For machine learning-based methods, we included RandomForest [16], a popular embedded feature selection method, and RelieF [17], a filter-based feature selection method. Both methods produce ranking of genes for their utility/importance in discriminating cell types.

### Performance evaluation

#### Cell type classification evaluation metrics

For each of all datasets sampled from the two atlases, we split 50% of a dataset into training data and the rest as test data. We applied different feature selection methods on training data to select marker genes of each cell type and then took the union of the top-5, 10, and 20 marker genes from each of all cell types for cell type classification on the test data using standard KNN (*k* = 7) and SVM classifiers. For the colon tissue datasets, samples from healthy individuals were randomly split into training and test sets. Feature selections were conducted on training data and the union of the top-10 marker genes from each of all cell types was used for cell type classification on test sets using a standard KNN (*k* = 7) classifier. Specifically, we used a “one versus all” approach by classifying cells from each cell type against cells from other cell types and calculating true positive (TP), false positive (FP), true negative (TN), and false negative (FN) for each cell type. These four quantities allowed us to compute F1 score, sensitivity, and specificity as follows

$$\begin{array}{c}F1\:score=2TP/(2TP+FP+FN)\\Sensitivity=TP/(TP+FN)\\Specificity=TN/(TN+FP)\end{array}$$

Lastly, we evaluated the performance of each classification model by calculating the mean F1 score, sensitivity, and specificity as the sum of each metric across all cell types divided by the number of cell types in a dataset.

#### Reproducibility evaluation metric

To quantify the reproducibility of a feature selection method, for each feature selection method, we calculated the pairwise Pearson’s correlation coefficient of gene ranks from datasets containing the same cell types but randomly sampled cells (see the “Reproducibility assessment by random sampling of cells” section for details on data sampling). To assess the reproducibility across different feature selection methods, we took either top-500 or top-1000 highly ranked genes from each method and quantified the degrees of overlaps of these top-ranked genes using Jaccard index.

#### Evaluation on tissue condition classification

The colon tissue profiling datasets were used for evaluating the utility of selected genes on classifying tissue conditions. For each of the three datasets that contain stromal, immune, and epithelial cells from all healthy individuals, we applied different feature selection methods to select marker genes. Next, following the design in a previous study on disease outcome prediction using scRNA-seq data [33], for each cell type, we created pseudo-bulk expression matrix based on their tissue conditions (i.e., healthy and inflamed) and evaluated the top-10 marker genes on classifying tissue condition using a standard KNN (*k* = 7) classifier and a fivefold cross-validation procedure to split the pseudo-bulk samples and reported the mean F1 scores.

#### Hardware specification for computational time

The computational time of differential distribution-based and machine-learning-based feature selection methods was recorded on an Oracle cloud instance with AMD OCPU (32 cores) and 512 GB RAM. In particular, RelieF, Wilcoxon, DESeq2, and Limma-voom only use one CPU whereas scDD and RandomForest which perform multi-threading were set to use 24 cores. Since deep learning-based feature selection algorithms largely rely on GPU in their computing, we recorded computational time for these methods on a locally configured machine with an RTX3090 GPU and an AMD Ryzen 5950x (16 cores) and 128 GB RAM. To test deep learning-based methods without GPU, we also recorded computational time for these methods on the same Oracle cloud instance that performed distribution-based and machine-learning-based feature selection methods. For all methods, time used for loading data was included. For deep learning-based methods, time for training the neural network model was also included.

### Supplementary Information

**Additional file 1: Fig S1.** Performance of feature selection methods for cell type classification using SVM  on datasets sampled from Tabula Muris atlas. **Fig. S2.** Performance of feature selection methods for cell type classification using KNN  on datasets sampled from Tabula Sapiens atlas. **Fig S3.** Summary of cell type classification F1 scores on Tabula Muris and Tabula  Sapiens atlases using genes selected by different feature selection methods. **Fig S4.** Impact of number of cells on cell type classification using the union of top-10 cell  type marker genes selected by each feature selection method. **Fig S5.** Impact of number of cells on cell type classification. **Fig S6.** F1  scores of KNN classification on minor cell types from datasets with imbalance ratios  of number of cells from major and minor cell type set as 2:1, 4:1, and 10:1, and numbers of cell  types set as 10 and 20. Each setting was repeated 10 times by random sampling from Tabula  Sapiens atlas for evaluating variability in performance. **Fig S7.** The rank of median F1 scores on datasets sampled from Tabula Muris and Tabula  Sapiens atlases with respect to different values of imbalance ratio. **Fig S8.** Reproducibility of feature selection results across methods. **Fig S9.** Expression profiles of marker genes selected by different feature selection  methods from a representative dataset sampled from Tabula Muris atlas. **Fig S10.** Computational time of feature selection methods on datasets with the number of cells increases from 50 to 250 with an increment of 50 and the number of cell types held as 10. The deep learning-based methods were evaluated using GPU and CPU configurations respectively. **Fig S11.** Characteristics of genes selected by different methods. **Fig S12.** Performance of genes selected by different methods in classifying granular cell  types and in tissue conditions. **Fig S13.** Evaluation of cell type classification without (i.e. all genes) and with feature  selections using Limma-voom and DeepLIFT on Tabula Muris and Tabula Sapiens  atlases.

**Additional file 2.** Review History.

## Data Availability

Data used in this study were obtained from the Tabula Muris project [18] (https://tabula-muris.ds.czbiohub.org/), Tabula Sapiens project [19] (https://tabula-sapiens-portal.ds.czbiohub.org/), and the Single Cell Portal with accession ID SCP259 [20] (https://singlecell.broadinstitute.org/single_cell/study/SCP259). Deep learning feature selection methods were implemented in Pytorch (1.11.0) and the source code is deposited in Zenodo (https://doi.org/10.5281/zenodo.10027186) [34] and is freely available from https://github.com/PYangLab/scDeepFeatures under a GPL-3 license [35].
