# Supplementary material for: Evaluation of deep learning-based feature selection for single-cell RNA sequencing data analysis
Source: Genome Biol. 2023 Nov 10;24:259. doi: 10.1186/s13059-023-03100-x (PMC10638755; doi:10.1186/s13059-023-03100-x)
Supplement: Supplementary file 1 — Additional file 1: Fig S1. Performance of feature selection methods for cell type classification using SVM on datasets sampled from Tabula Muris atlas. Fig. S2. Performance of feature selection methods for cell type classification using KNN on datasets sampled from Tabula Sapiens atlas. Fig S3. Summary of cell type classification F1 scores on Tabula Muris and Tabula Sapiens atlases using genes selected by different feature selection methods. Fig S4. Impact of number of cells on cell type classification using the union of top-10 cell type marker genes selected by each feature selection method. Fig S5. Impact of number of cells on cell type classification. Fig S6. F1 scores of KNN classification on minor cell types from datasets with imbalance ratios of number of cells from major and minor cell type set as 2:1, 4:1, and 10:1, and numbers of cell types set as 10 and 20. Each setting was repeated 10 times by random sampling from Tabula Sapiens atlas for evaluating variability in performance. Fig S7. The rank of median F1 scores on datasets sampled from Tabula Muris and Tabula Sapiens atlases with respect to different values of imbalance ratio. Fig S8. Reproducibility of feature selection results across methods. Fig S9. Expression profiles of marker genes selected by different feature selection methods from a representative dataset sampled from Tabula Muris atlas. Fig S10. Computational time of feature selection methods on datasets with the number of cells increases from 50 to 250 with an increment of 50 and the number of cell types held as 10. The deep learning-based methods were evaluated using GPU and CPU configurations respectively. Fig S11. Characteristics of genes selected by different methods. Fig S12. Performance of genes selected by different methods in classifying granular cell types and in tissue conditions. Fig S13. Evaluation of cell type classification without (i.e. all genes) and with feature selections using Limma-voom and DeepLIFT on Tabula Muris and Tab [file 13059_2023_3100_MOESM1_ESM.pdf]

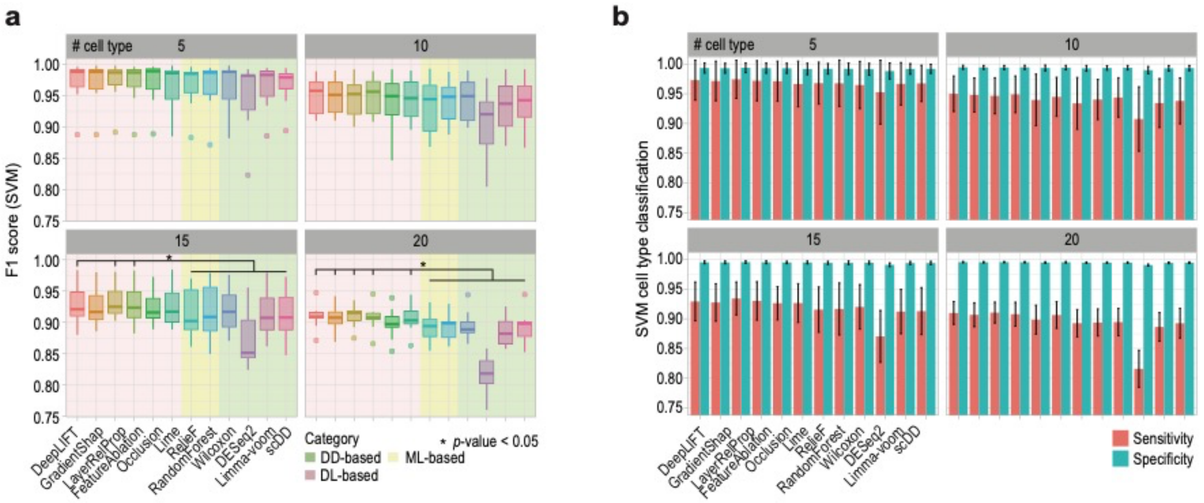

Fig S1. Performance of feature selection methods for cell type classification using SVM on datasets sampled from Tabula Muris atlas. (**a**) F1 scores of cell type classification using SVM and the union of top-10 genes selected from each cell type by each feature selection method. The number of cell types varies from 5 to 20. The process was repeated 10 times by random sampling from Tabula Muris atlas. Paired Wilcox rank sum tests were performed to evaluate the statistical significance. (**b**) Similar to (**a**) but quantifying classification performance using sensitivity and specificity.

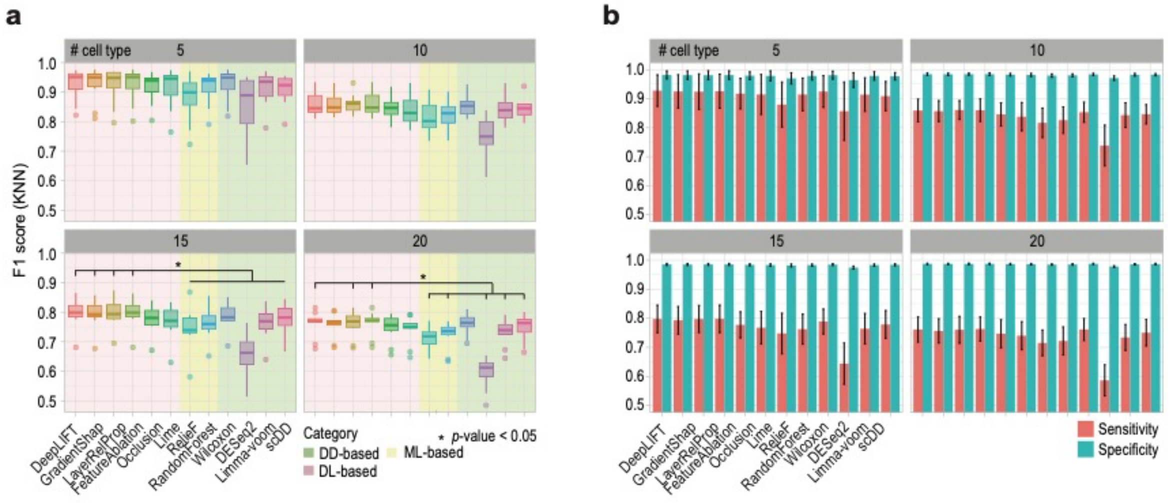

Fig S2. Performance of feature selection methods for cell type classification using KNN on datasets sampled from Tabula Sapiens atlas. (**a**) F1 scores of cell type classification using KNN and the union of top-10 genes selected from each cell type by each feature selection method. The number of cell types varies from 5 to 20. The process was repeated 10 times by random sampling from Tabula Sapiens atlas. Paired Wilcox rank sum tests were performed to evaluate the statistical significance. (**b**) Similar to (**a**) but quantifying classification performance using sensitivity and specificity.

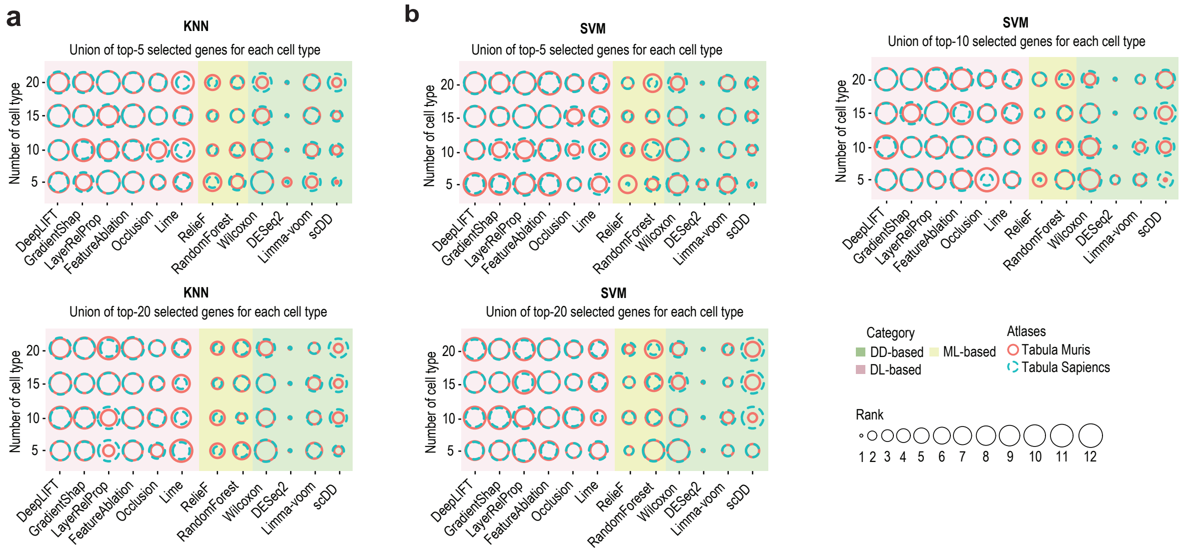

Fig S3. Summary of cell type classification F1 scores on Tabula Muris and Tabula Sapiens atlases using genes selected by different feature selection methods. (**a**) For numbers of cell types set at 5, 10, 15, and 20, each balloon plots summarising the ranks of median F1 scores from KNN on datasets sampled from Tabula Muris and Tabula Sapiens atlases. Classification was performed using either union of top-5 or top-20 genes selected from each cell type by each feature selection method. The size of the balloon represents the rank of the method, the larger the better its performance. (**b**) Similar to (**a**) but using SVM classifier for cell type classification. Panels present results from using union of top-5, top-10, or top-20 genes selected from each cell type by each feature selection method.

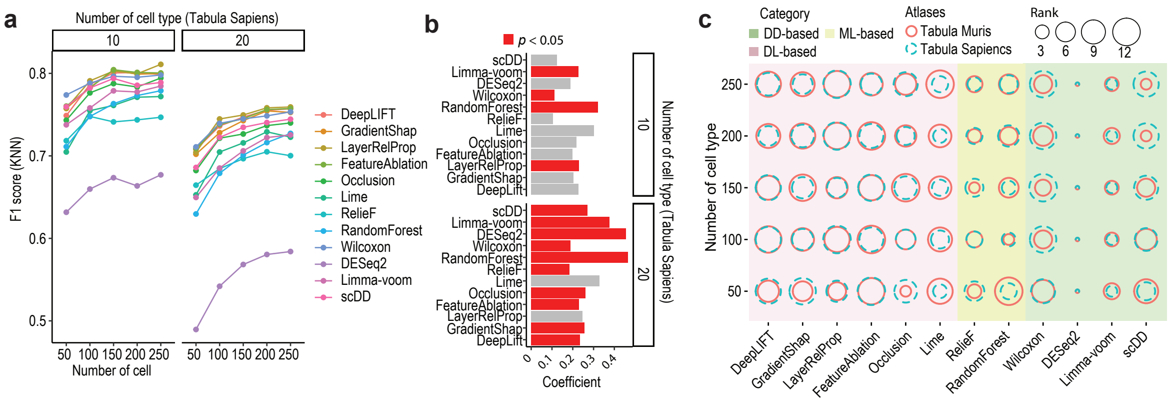

Fig S4. Impact of number of cells on cell type classification using the union of top-10 cell type marker genes selected by each feature selection method. (**a**) Mean F1 scores of KNN classification of 10 or 20 cell types (sampled from Tabula Sapiens atlas) each with number of cells set as 50, 100, 150, 200, and 250. (**b**) Coefficients of slopes from least squares fitted lines to median F1 scores in (**a**) across number of cells. (**c**) For numbers of cells set as 50, 100, 150, 200, and 250 and number of cell types fixed at 20, balloon plots summarising the ranks of median F1 scores from SVM on datasets sampled from Tabula Muris and Tabula Sapiens atlases. The size of the balloon represents the rank of the method, the larger the better its performance.

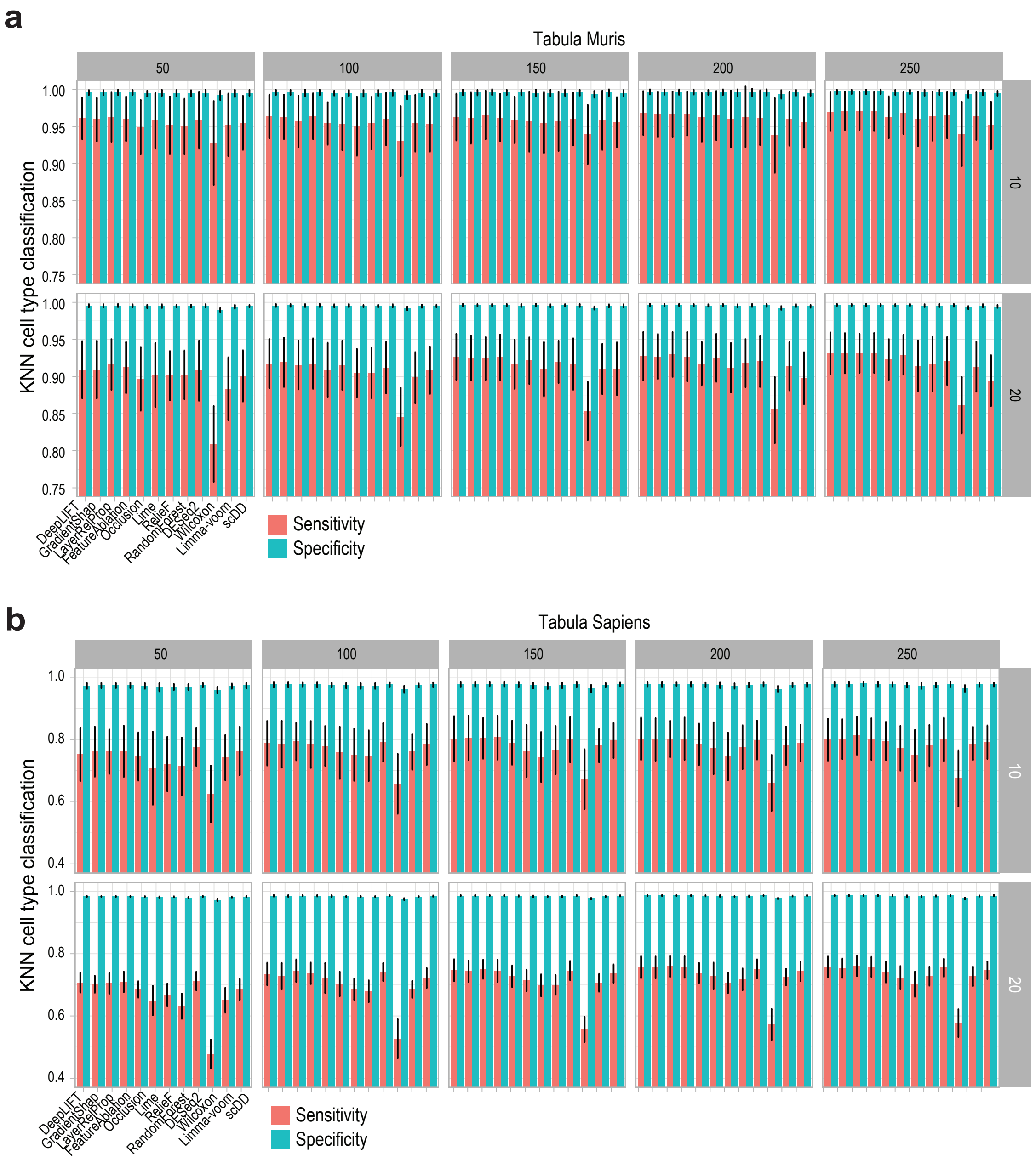

Fig S5. Impact of number of cells on cell type classification. Classification accuracy of KNN quantified using sensitivity and specificity on datasets sampled from (**a**) Tabula Muris and (**b**) Tabula Sapiens atlases. Number of cells in each cell type increases from 50 to 250 with an increment of 50 and the number of cell types held at 10 and 20, respectively.

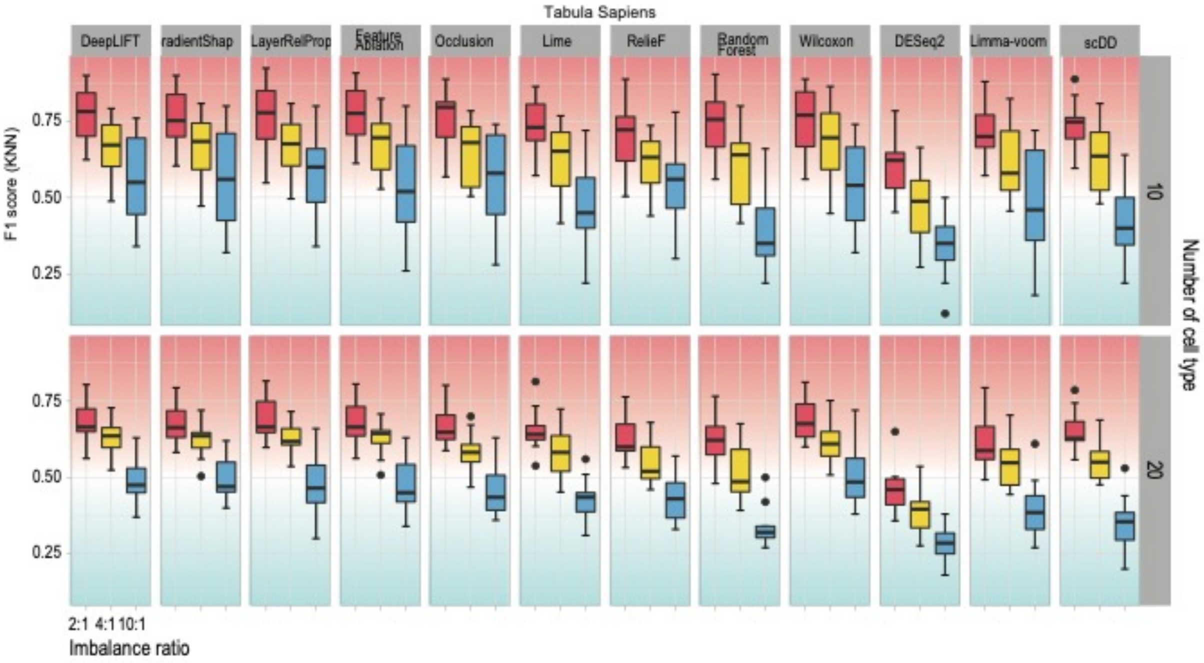

Fig S6. F1 scores of KNN classification on minor cell types from datasets with imbalance ratios of number of cells from major and minor cell type set as 2:1, 4:1, and 10:1, and numbers of cell types set as 10 and 20. Each setting was repeated 10 times by random sampling from Tabula Sapiens atlas for evaluating variability in performance.

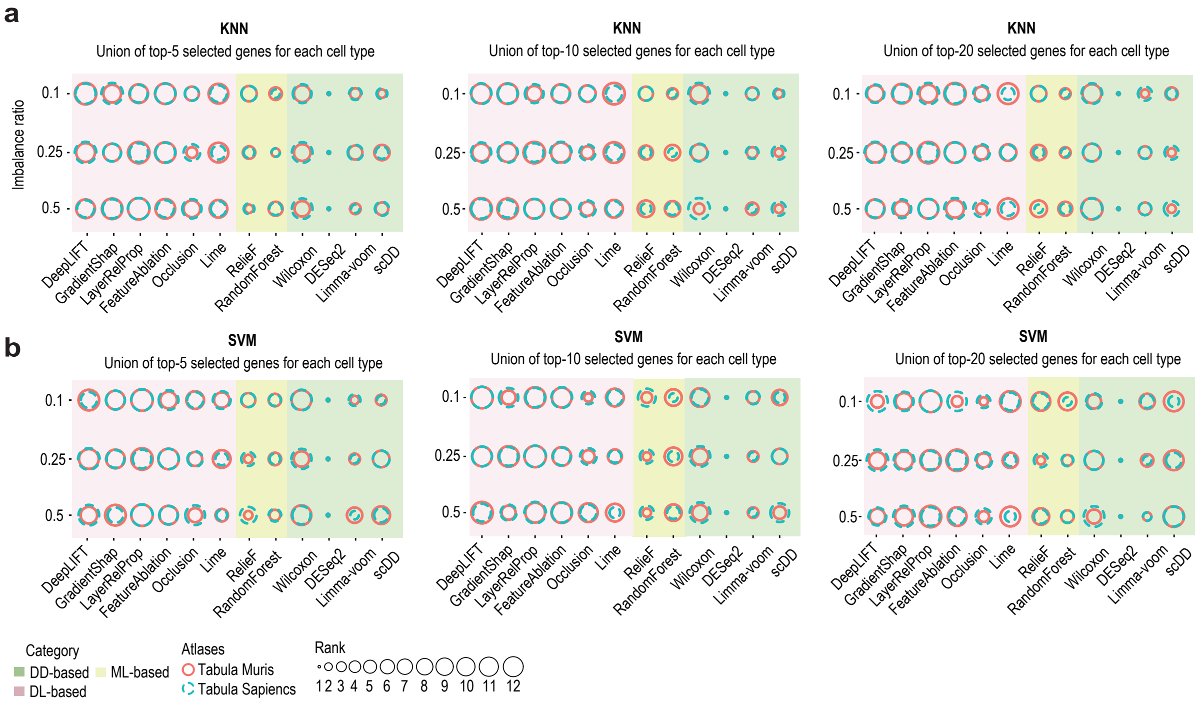

Fig S7. The rank of median F1 scores on datasets sampled from Tabula Muris and Tabula Sapiens atlases with respect to different values of imbalance ratio. The number of cell types were fixed as 20. (**a**) The rank of median F1 scores based on KNN classification with the union of top 5, 10, and 20 marker genes of each cell type. (**b**) The rank of median F1 scores based on SVM classification, with the union of top 5, 10, 20 marker genes of each cell type.

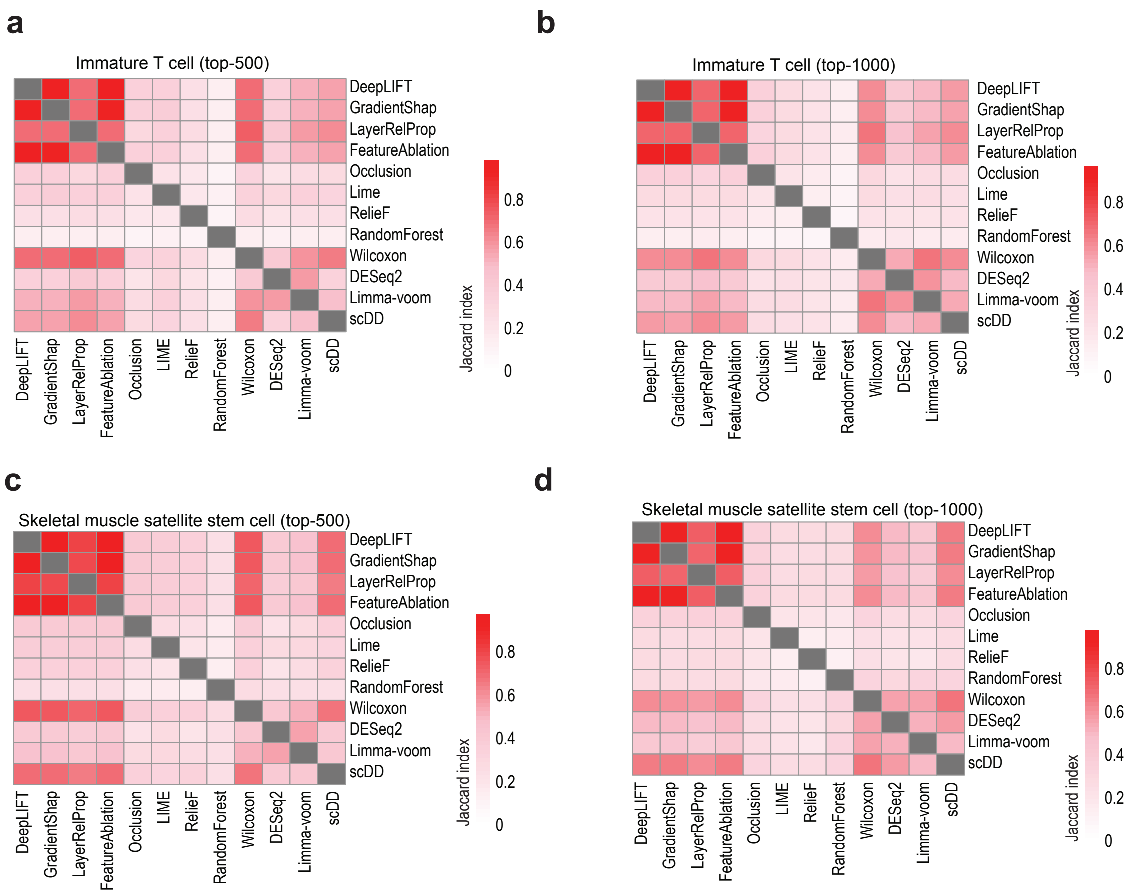

Fig S8. Reproducibility of feature selection results across methods. (**a**, **b**) Pairwise overlaps of top-500 and top-1000 genes selected by each method for Immature T cell type as quantified by Jaccard index. (**c**, **d**) Pairwise overlaps of top-500 and top-1000 genes selected by each method for Skeletal muscle satellite stem cell type as quantified by Jaccard index.

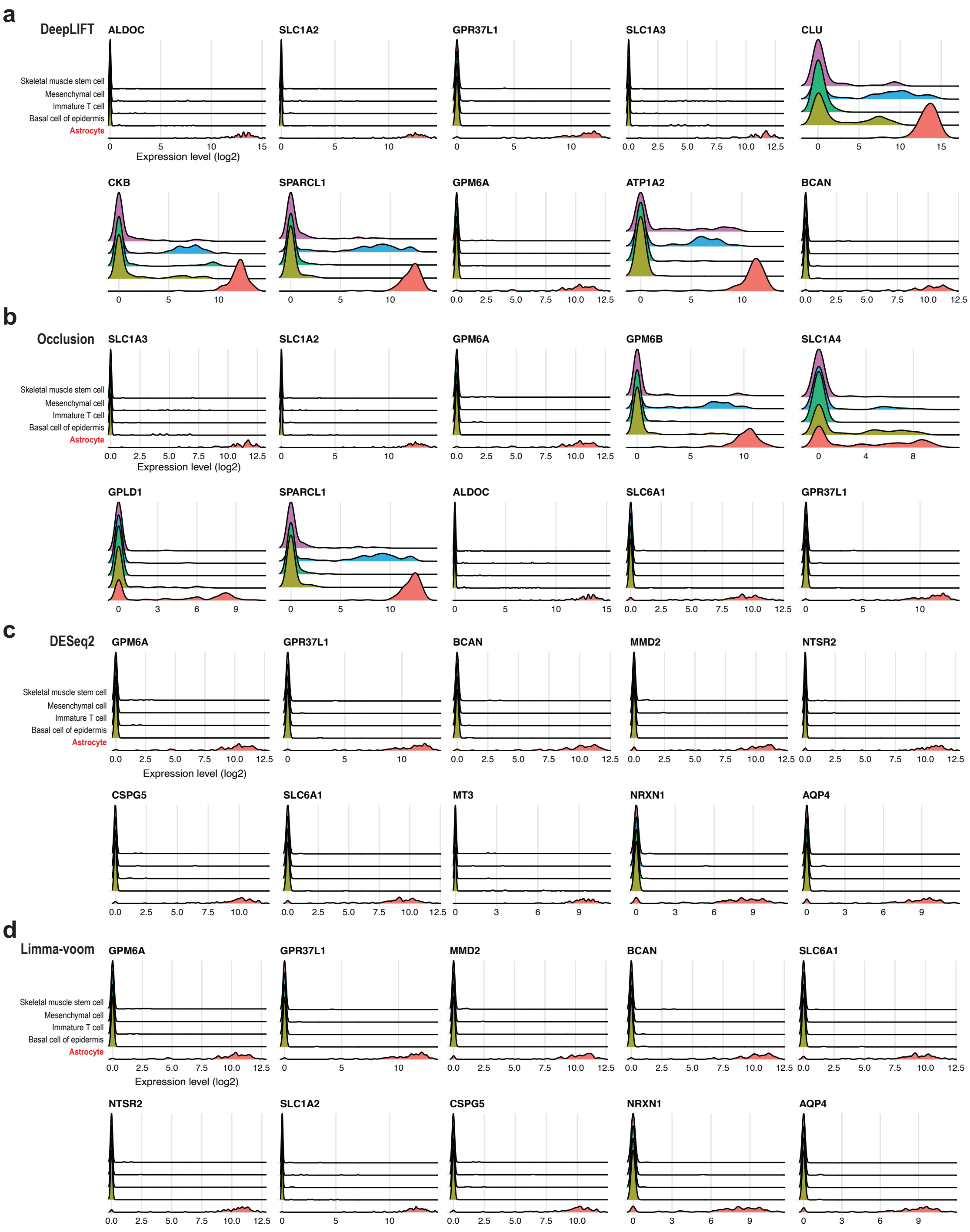

Fig S9. Expression profiles of marker genes selected by different feature selection methods from a representative dataset sampled from Tabula Muris atlas. Expression profiles of top-10 marker genes for “Astrocyte” cell type selected by two deep learning-based methods, (**a**) DeepLIFT and (**b**) Occlusion, and two differential distribution-based methods, (**c**) DESeq2 and (**d**) Limma-voom are shown.

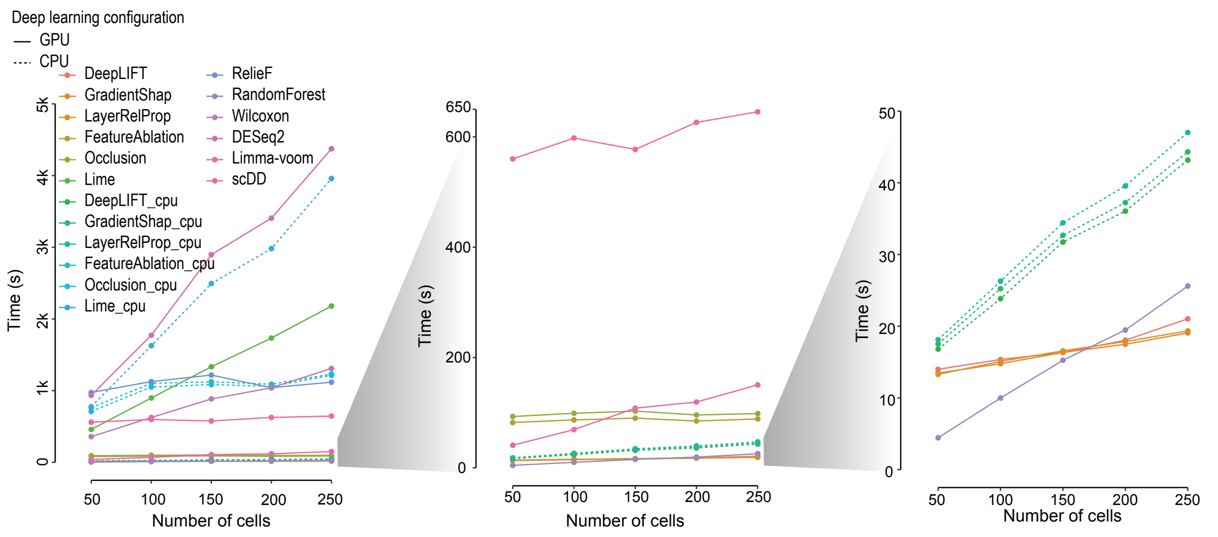

Fig S10. Computational time of feature selection methods on datasets with the number of cells increases from 50 to 250 with an increment of 50 and the number of cell types held as 10. The deep learning-based methods were evaluated using GPU and CPU configurations respectively.

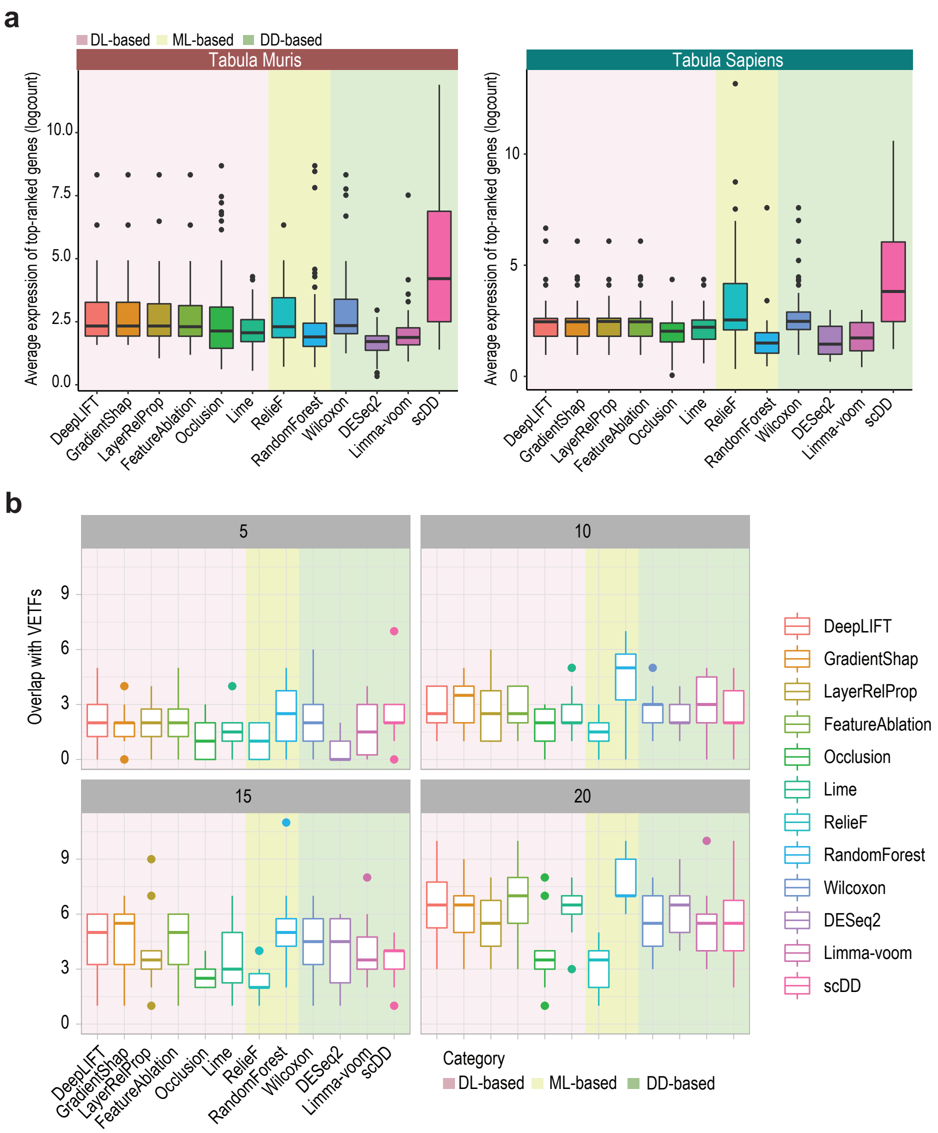

Fig S11. Characteristics of genes selected by different methods. (**a**) Average expression levels of top-10 selected genes by different methods in datasets subsampled from Tabula Muris and Tabula Sapiens atlases. (**b**) Overlap between the top-10 selected genes by different methods and the variably expressed transcription factors (VETFs) that mark cell types.

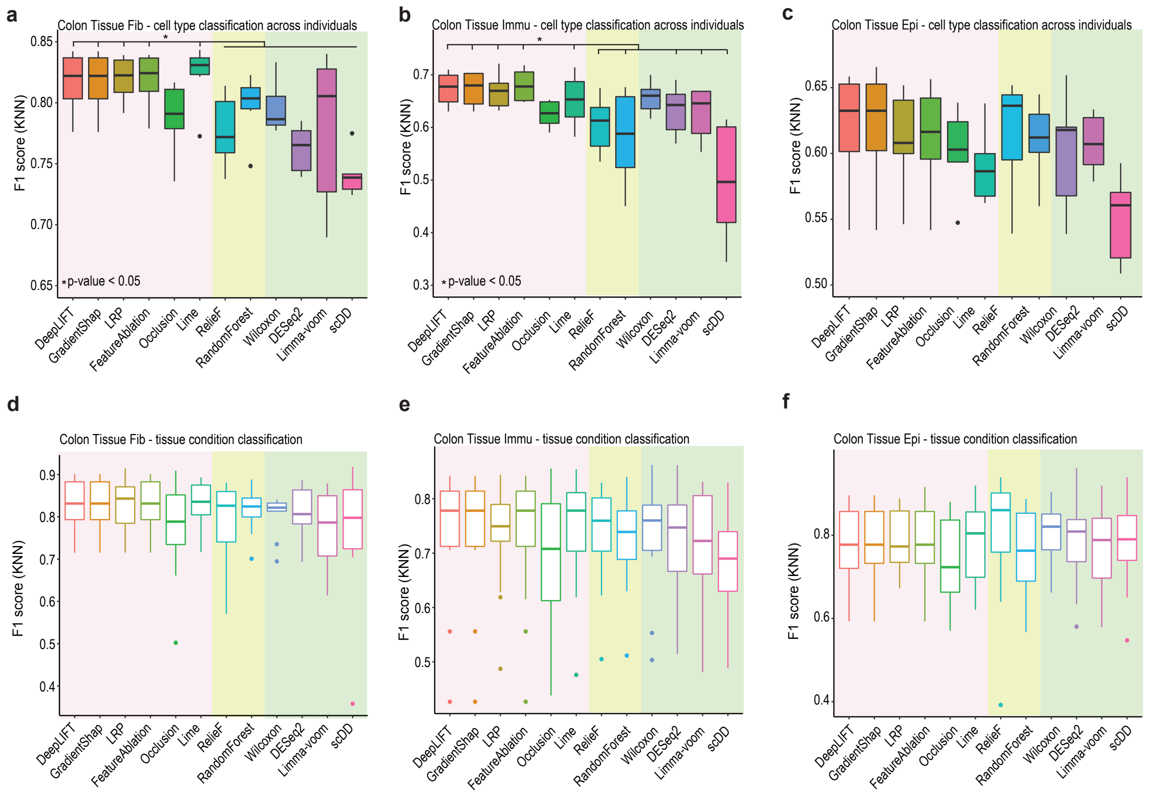

Fig S12. Performance of genes selected by different methods in classifying granular cell types and in tissue conditions. (**a-c**) F1 scores of top-10 selected genes by each feature selection method for classifying granular cell types grouped by three main cell type categories including Fib, Immu, and Epi, and sampled from healthy individuals. (**d-f**) F1 scores of top-10 selected genes from the three main cell type categories for classifying tissue conditions.

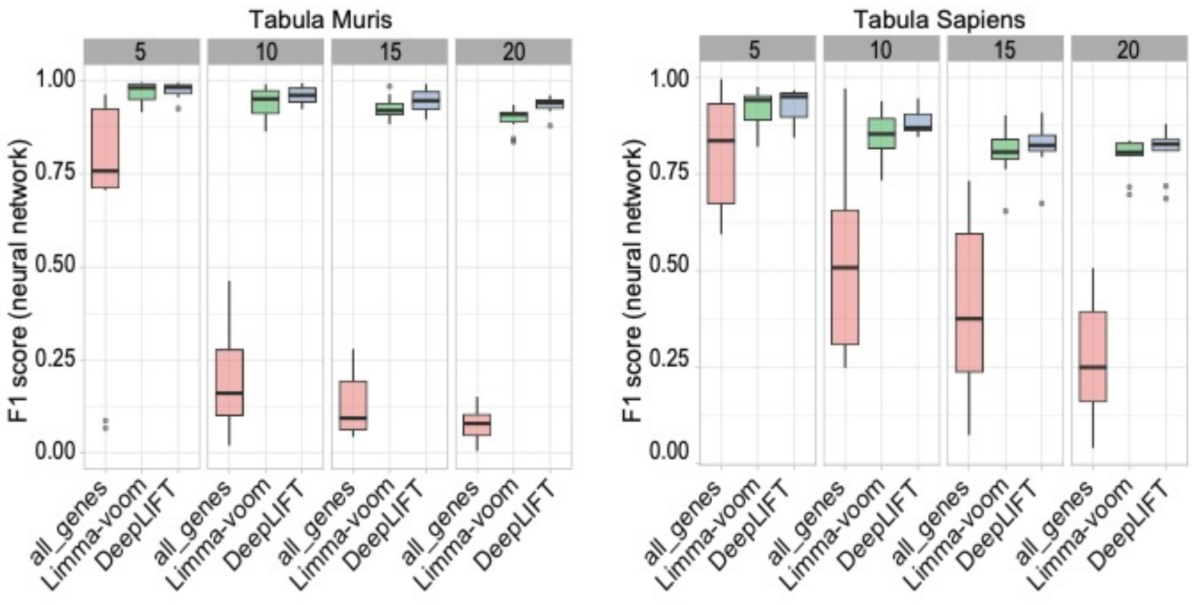

Fig S13. Evaluation of cell type classification without (i.e. all genes) and with feature selections using Limma-voom and DeepLIFT on Tabula Muris and Tabula Sapiens atlases.
