# Supplementary material for: Evaluation of deep learning-based feature selection for single-cell RNA sequencing data analysis
Source: Genome Biol. 2023 Nov 10;24:259. doi: 10.1186/s13059-023-03100-x (PMC10638755; doi:10.1186/s13059-023-03100-x)
Supplement: Supplementary file 2 — Additional file 2. Review History. [file 13059_2023_3100_MOESM2_ESM.docx]

Review History

**First round of review**

**Reviewer 1**

**Are you able to assess all statistics in the manuscript, including the appropriateness of statistical tests used?** Yes, and I have assessed the statistics in my report.

**Comments to author:**

The manuscript by Huang et al. entitled "Evaluation of deep learning-based feature selection for single-cell RNA sequencing data analysis" describes a comparison of the performance of several deep learning feature selection methods (both gradient back-propagation and feature perturbation approaches) in comparison with more traditional statistical differential gene expression methods in terms of cell type classification, feature selection reproducibility and diversity, and computational time using two scRNA-seq datasets - Tabula Muris and Tabula Sapiens. They find that the machine learning-based methods generally outperform the more traditional statistical methods by each of these evaluation criteria.

Feature selection in the setting of scRNA-seq data analysis is indeed important as a type of dimensionality reduction that can help overcome the so-called "curse of dimensionality", which makes it difficult to obtain optimal classification results when the feature space is too high. Any method that can select informative features for improved cell type classification would be valuable. In general, the evaluation approach reported here in which features are selected from deep neural network models or differential expression statistical tests and evaluated for their performance in building cell type classification models based on KNN and SVM is appropriate for this purpose.

Three major concerns are noted:

First, if I am understanding the approaches correctly, I would argue that the gradient back-propagation and feature perturbation approaches used are not actually performing "feature selection" perse but are rather identifying features that have already been deemed to be informative by the neural network model. The authors used a simple multilayer perceptron (MLP) approach to build classification models for the evaluation of all deep learning-based "feature selection" approaches. Thus, the deep learning-based "feature selection" approaches are being used to identify the features that have actually been selected by the MLP neural network to output the probability vector of cell type predictions. This is more than an issue of semantics. It would explain why there might be relatively high overlap (reproducibility) in the features obtained by the deep learning feature selection methods, as seen in the Jaccard index values reported in Figure 5, since they are all trying to extract the same set of informative features from the same neural network models. This nuance needs to be discussed in the manuscript.

Second, this means that they are only comparing the single machine-learning based feature selection approach implemented in the MLP. This work would have a greater impact if they were to compare the traditional statistical differential expression approaches with at least one other machine learning method. For example, random forest machine learning outputs ranked lists of informative features based on Gini index or information gain. It would be useful to see if higher performance is a general finding of ML-based feature selection.

Third, the scRNA-seq datasets used in this study (Tabula Muris and Tabula Sapiens) do not represent the state of the art in cell type classification from scRNA-seq transcriptional profiling. Once the authors performed some basic quality control and abundance threshold filtering on the two datasets, they ended up with only 38 cell types in the entire Tabula Muris atlas and only 30 cell types in the entire Tabula Sapiens atlas. However, the combined analysis of more comprehensive collections in resources like cellxgene (https://cellxgene.cziscience.com/collections) suggest there are likely thousands of distinct cell types in humans. The two Tabula datasets used here likely only capture very course cell type distinctions for cell populations that are relatively abundant. It would be important to perform this evaluation on datasets with more granular cell types defined or by reducing the abundance threshold to include rarer cell types in the analysis.

**Reviewer 2**

**Are you able to assess all statistics in the manuscript, including the appropriateness of statistical tests used?** There are no statistics in the manuscript.

**Comments to author:**

Summary

The authors compare several feature selection methods in single-cell RNA-seq analysis. Notably, the methods used in deep learning and regulatory genomics communities were considered in addition to traditional feature selection methods based on gene-by-gene statistics. The authors measured the performance by cell type classification accuracy, reproducibility, and computation time in two large-scale data sets.

Major points

Overall, the paper is well-written, definitely strong, and address an important problem in single-cell data analysis.

1. The paper would have been more impactful if the feature selection tasks were used beyond cell type classification problems, such as case-control studies or disease progression prediction. A common practice of single-cell RNA-seq analysis proceeds with unsupervised learning followed by cell type assignment for each cell cluster.

2. I recommend the authors consider different evaluation schemes. Cell type definitions in Tabula Spaiens and Tabula Muris were actually the outcome of latent embedding (e.g., variational autoencoders, unsupervised DL) based on top 500 or 5000 features and graph-based clustering. DL-based methods based on non-linear, intrinsic feature engineering clearly take advantage with little risk of over-fitting problems. Therefore, it is no surprise to see such high specificity scores regardless of the methods. It wouldn’t be surprising to see that the top 500 or 5000 genes are also attainable for high F1 scores as they were used in initial cell type clustering analysis.

3. Need more background knowledge and details on deep-learning-based feature selection

methods: I think we can think of two ways of doing DL-based feature selection methods in supervised and unsupervised/self-supervised learning. I assume the authors used the feature selection methods after deep neural networks were trained in supervised learning, predicting cell types based on high-dimensional gene expression vectors.

4. How does a NN classifier without any feature selection perform? I wonder if there were enough merit in feature selection methods in a cell type classification task.

5. Statistical methods provide scores pertinent to error probabilities (Type-I), which can be directly translated to false discovery rates. I also wonder if DL-based methods can provide such a way to handle uncertainties.

Minor points

• Comparing GPU and CPU time is not fair. CPU-based methods were not parallelized although they can be embarrassingly parallel.

• Did we find any new biological insights using DL-based methods?

**Reviewer 3**

**Are you able to assess all statistics in the manuscript, including the appropriateness of statistical tests used?** Yes, and I have assessed the statistics in my report.

**Comments to author:**

The manuscript provides a comparison of deep learning-based feature selection methods and statistical models for identifying differential gene expression in scRNA-seq data, with a focus on cell type classification. While the study is interesting and explores the utility of deep learning methods for this purpose, there are several important issues that need to be addressed to ensure the validity of the conclusions.

Major Points:

1. The manuscript compares deep learning-based methods and statistical models based on their performance in cell type classification. However, the evaluation design gives an advantage to the deep learning methods, as they are trained on one half of the data and tested on the other half, which is very similar. On the other hand, statistical methods that identify genes with significant expression differences between cell types are not designed for cell type discrimination. This design may lead to overfitting of the deep learning methods and the gene lists generated may not be generalizable to independent datasets with similar cell types. A fairer and more practical comparison would be to evaluate the quality of the selected gene lists on an independent dataset instead of splitting the data into training and test sets. This will provide insights into whether deep learning methods truly have an advantage in a more realistic setting.

2. Since the deep learning methods and statistical models select different sets of genes, it would be helpful to conduct a more detailed analysis to understand the reasons behind these differences. The authors should explore and discuss the characteristics of the selected genes, such as whether they are known cell type marker genes and how their expression levels compare to average gene expressions. This analysis will provide a deeper understanding of the differences between the methods.

3. (a) More details should be provided on how the deep learning methods and MLP (multi-layer perceptron) were set up for feature selection in the context of scRNA-seq data, including the process of extracting importance scores. (b) Some notations used in the description of the deep learning methods are unclear and should be clearly defined for better understanding.

4. In the section on "Feature selection reproducibility within and across feature selection methods," the authors should provide further justification for why having a diversified expression in the selected genes is desirable. Additional explanation will help readers understand the rationale behind this criterion.

5. The manuscript does not include some popular statistical methods for scRNA-seq analysis, such as the Wilcoxon test used in the widely-used scRNA-seq package Seurat. Considering the popularity of these methods, it would be valuable to include them in the comparison to provide a comprehensive evaluation.

**Authors Response**

**Point-by-point responses to the reviewers’ comments:**

Reviewer 1:

The manuscript by Huang et al. entitled "Evaluation of deep learning-based feature selection for single-cell RNA sequencing data analysis" describes a comparison of the performance of several deep learning feature selection methods (both gradient back-propagation and feature perturbation approaches) in comparison with more traditional statistical differential gene expression methods in terms of cell type classification, feature selection reproducibility and diversity, and computational time using two scRNA-seq datasets - Tabula Muris and Tabula Sapiens. They find that the machine learning-based methods generally outperform the more traditional statistical methods by each of these evaluation criteria.

Feature selection in the setting of scRNA-seq data analysis is indeed important as a type of dimensionality reduction that can help overcome the so-called "curse of dimensionality", which makes it difficult to obtain optimal classification results when the feature space is too high. Any method that can select informative features for improved cell type classification would be valuable. In general, the evaluation approach reported here in which features are selected from deep neural network models or differential expression statistical tests and evaluated for their performance in building cell type classification models based on KNN and SVM is appropriate for this purpose.

Response: We thank the reviewer for affirming the importance of the feature selection in single-cell data analysis and the positive comments on the appropriateness of our analyses.

Three major concerns are noted:

1. First, if I am understanding the approaches correctly, I would argue that the gradient back-propagation and feature perturbation approaches used are not actually performing "feature selection" perse but are rather identifying features that have already been deemed to be informative by the neural network model. The authors used a simple multilayer perceptron (MLP) approach to build classification models for the evaluation of all deep learning-based "feature selection" approaches. Thus, the deep learning-based "feature selection" approaches are being used to identify the features that have actually been selected by the MLP neural network to output the probability vector of cell type predictions. This is more than an issue of semantics. It would explain why there might be relatively high overlap (reproducibility) in the features obtained by the deep learning feature selection methods, as seen in the Jaccard index values reported in Figure 5, since they are all trying to extract the same set of informative features from the same neural network models. This nuance needs to be discussed in the manuscript.

Response: We thank the reviewer for this insight. The reviewer is correct that the neural network model intrinsically learns the informativeness of input features. However, additional feature selection techniques such as these evaluated in this current study are required to extract such information from the model. We acknowledge this may have led to higher overlap among different feature selection methods implemented for the neural network model. As suggested, we have now clarified and discussed this in the revised manuscript (second paragraph of the “Introduction” section and second paragraph of the “Discussion and conclusion” section).

2. Second, this means that they are only comparing the single machine-learning based feature selection approach implemented in the MLP. This work would have a greater impact if they were to compare the traditional statistical differential expression approaches with at least one other machine learning method. For example, random forest machine learning outputs ranked lists of informative features based on Gini index or information gain. It would be useful to see if higher performance is a general finding of ML-based feature selection.

Response: We thank the reviewer for the valuable suggestion. We have now included the Random Forest algorithm for feature selection in our experiments. We have also included RelieF, another commonly employed feature selection algorithm from computer science and machine learning literature for comparison given its popularity and reported high performance (PMID: 30031057). We have now updated all benchmark experiments to include results from these new methods.

3. Third, the scRNA-seq datasets used in this study (Tabula Muris and Tabula Sapiens) do not represent the state of the art in cell type classification from scRNA-seq transcriptional profiling. Once the authors performed some basic quality control and abundance threshold filtering on the two datasets, they ended up with only 38 cell types in the entire Tabula Muris atlas and only 30 cell types in the entire Tabula Sapiens atlas. However, the combined analysis of more comprehensive collections in resources like cellxgene (https://protect-au.mimecast.com/s/u1_pCWLVXkU6Npg3JUmxwJ8?domain=cellxgene.cziscience.com) suggest there are likely thousands of distinct cell types in humans. The two Tabula datasets used here likely only capture very course cell type distinctions for cell populations that are relatively abundant. It would be important to perform this evaluation on datasets with more granular cell types defined or by reducing the abundance threshold to include rarer cell types in the analysis.

Response: We thank the reviewer for this comment. As suggested, we have now included another large collection of scRNA-seq data generated from profiling human tissues and with cell type grouped into three large categories (epithelial cells, stromal cells, and immune cells) each with more granular cell type annotations (PMID: 31348891). We found that the evaluation of feature selection methods on these newly added datasets are consistent with those from Tabula Muris and Tabula Sapiens datasets and further strengthened the results. We have now included these results in the new Supplementary Figure 12a and revised the manuscript accordingly to incorporate these new results (section “Characteristics of selected genes and further evaluation on scRNA-seq data with granular cell types and across samples”).

Reviewer 2:

The authors compare several feature selection methods in single-cell RNA-seq analysis. Notably, the methods used in deep learning and regulatory genomics communities were considered in addition to traditional feature selection methods based on gene-by-gene statistics. The authors measured the performance by cell type classification accuracy, reproducibility, and computation time in two large-scale data sets.

Major points

Overall, the paper is well-written, definitely strong, and address an important problem in single-cell data analysis.

Response: We thank the reviewer for the positive comments on our work and the importance of the problem we are addressing. We have taken into consideration all suggested points below.

1. The paper would have been more impactful if the feature selection tasks were used beyond cell type classification problems, such as case-control studies or disease progression prediction. A common practice of single-cell RNA-seq analysis proceeds with unsupervised learning followed by cell type assignment for each cell cluster.

Response: We thank the reviewer for this suggestion. We have now included another large collection of scRNA-seq data generated from profiling colon tissues obtained from healthy individuals and ulcerative colitis patients and tested the utility of selected genes on classifying tissue disease status (i.e., a case-control setting). While the performance of genes selected by different methods appears to be comparable to each other, these results demonstrate that selected genes can be used for discriminating samples based on their disease status. These new results are now included in the Supplementary Figure 12b in the revised manuscript.

2. I recommend the authors consider different evaluation schemes. Cell type definitions in Tabula Spaiens and Tabula Muris were actually the outcome of latent embedding (e.g., variational autoencoders, unsupervised DL) based on top 500 or 5000 features and graph-based clustering. DL-based methods based on non-linear, intrinsic feature engineering clearly take advantage with little risk of over-fitting problems. Therefore, it is no surprise to see such high specificity scores regardless of the methods. It wouldn’t be surprising to see that the top 500 or 5000 genes are also attainable for high F1 scores as they were used in initial cell type clustering analysis.

Response: We appreciate these comments. Indeed, we assessed the performance of different methods in terms of cell type classification, feature selection reproducibility, and computational cost. As recommended in point 1, we have now also extended the assessment of the performance of methods on a case-control setting. As for cell type annotation, the Tabula Muris project used a standard pipeline of filtering, variable gene selection, PCA projection, clustering of PCA projected cells, and finally annotation of cell types using known marker genes (PMID: 30283141); and the Tabula Sapiens project formed an expert panel to annotate the cell types (PMID: 35549404). Both are not based on deep learning generated embeddings for cell type annotation, and the high specificity of cell type classification is not only observed from deep learning-based methods but also differential distribution-based methods (see e.g., Figure 2b). The high specificity is likely due to the use of the “one versus all” approach in quantifying classification performance where the precision is high when a cell is classified to a cell type, yet discriminating all cells of a cell type from the rest of the cell types is challenging, leading to varying sensitivity and F1 scores that distinguish the performance of each method. We have now discussed this in the revised manuscript (Section “Performance of feature selection methods on cell type classification and impact of number of cell types”).

3. Need more background knowledge and details on deep-learning-based feature selection methods: I think we can think of two ways of doing DL-based feature selection methods in supervised and unsupervised/self-supervised learning. I assume the authors used the feature selection methods after deep neural networks were trained in supervised learning, predicting cell types based on high-dimensional gene expression vectors.

Response: We thank the reviewer for this suggestion. Indeed, we are using feature selection methods after supervised training of neural network models on predicting cell types based on high-dimensional gene expression vectors. As suggested, we have clarified this and have included additional background information and details on deep learning-based feature selection methods implemented in this study (second paragraph of the “Introduction” section and second paragraph of the “Discussion and conclusion” section).

4. How does a NN classifier without any feature selection perform? I wonder if there were enough merit in feature selection methods in a cell type classification task.

Response: We thank the reviewer for suggesting this baseline comparison. We have now tested classifying cell types using all genes (without feature selection) using a neural network classifier. We found that cell type classification without selecting genes led to significantly poorer performance compared to those from using informative genes selected by deep learning-based methods (e.g., DeepLIFT) and differential expression-based methods (e.g. Limma). We presented these results in Supplementary Figure 13.

5. Statistical methods provide scores pertinent to error probabilities (Type-I), which can be directly translated to false discovery rates. I also wonder if DL-based methods can provide such a way to handle uncertainties.

Response: We thank this suggestion. While statistical methods have the advantage of estimating uncertainties on selected genes for controlling false discovery rate (FDR), deep learning-based feature selection methods can also provide FDR estimation. One approach is to compute the importance scores of genes from datasets with original cell type labels perturbed randomly. We have now noted this in the revised manuscript (fourth paragraph, section “Discussion and conclusion”).

Minor points

• Comparing GPU and CPU time is not fair. CPU-based methods were not parallelized although they can be embarrassingly parallel.

Response: We thank this suggestion and have now included the computation time on CPU for all deep learning-based methods. These results are included in updated Figure 6 and Supplementary Figure 10 of the revised manuscript.

• Did we find any new biological insights using DL-based methods?

Response: We thank this question. While the current work did not investigate the biology of the selected genes, we have now performed additional experiments to explore the characteristics of the gene selected by different methods and their utility beyond cell type classification. These new results are now included in the new section “Characteristics of selected genes and further evaluation on scRNA-seq data with granular cell types and across samples”.

Reviewer 3:

The manuscript provides a comparison of deep learning-based feature selection methods and statistical models for identifying differential gene expression in scRNA-seq data, with a focus on cell type classification. While the study is interesting and explores the utility of deep learning methods for this purpose, there are several important issues that need to be addressed to ensure the validity of the conclusions.

Response: We thank the reviewer for the constructive comments. We have taken onboard all suggestions from the reviewer.

Major Points:

1. The manuscript compares deep learning-based methods and statistical models based on their performance in cell type classification. However, the evaluation design gives an advantage to the deep learning methods, as they are trained on one half of the data and tested on the other half, which is very similar. On the other hand, statistical methods that identify genes with significant expression differences between cell types are not designed for cell type discrimination. This design may lead to overfitting of the deep learning methods and the gene lists generated may not be generalizable to independent datasets with similar cell types. A fairer and more practical comparison would be to evaluate the quality of the selected gene lists on an independent dataset instead of splitting the data into training and test sets. This will provide insights into whether deep learning methods truly have an advantage in a more realistic setting.

Response: We thank the reviewer for this comment. We note that while the deep learning-based methods (classified as embedded feature selection methods) selected genes based on a neural network model, both the deep learning-based methods and the differential distribution-based methods (classified as filter-based methods) we evaluated in this study selected genes that differentiate cell types (PMID: 34847932). The split of data for assessing the genes selected by different methods (embedded and filter methods in this case) on a training set in classifying cell types in a test set is a common practice and does not give an advantage to the deep learning methods as the gene selection step is independent from the evaluation of their utility in cell type classification. The evaluation on independent datasets is more informative for benchmarking data normalisation methods if batch effects and other unknown variants are present in the independent datasets. Nevertheless, we have now included additional experiments to evaluate these feature selection methods using datasets generated from a study that profiles the colon tissues from healthy individuals and patients where each individual can be considered as an independent sample that contains similar cell types (PMID: 31348891). These additional results further strengthen the comparisons of methods and are now included in a new section “Characteristics of selected genes and further evaluation on scRNA-seq data with granular cell types and across samples” in the revised manuscript.

2. Since the deep learning methods and statistical models select different sets of genes, it would be helpful to conduct a more detailed analysis to understand the reasons behind these differences. The authors should explore and discuss the characteristics of the selected genes, such as whether they are known cell type marker genes and how their expression levels compare to average gene expressions. This analysis will provide a deeper understanding of the differences between the methods.

Response: We thank this insightful comment. Our previous findings suggest that genes selected by deep learning-based methods appear to be more diverse in their expression profiles compared to those selected by differential expression-based methods. We have now discussed the rationale behind this finding (first paragraph, “Discussion and conclusion” section). In addition, we have now also explored the expression levels of the top-ranked genes and their overlap with the variably expressed transcription factors (VETF) defined in a previous study for marking cell types (PMID: 33278344). We found that genes selected by deep learning-based methods show relatively low to moderate expression levels compared to some traditional methods and have comparable numbers of overlaps with VETFs. These results are now presented in the new Supplementary Figure 11 and also the revised manuscript (new section “Characteristics of selected genes and further evaluation on scRNA-seq data with granular cell types and across samples”).

3. (a) More details should be provided on how the deep learning methods and MLP (multi-layer perceptron) were set up for feature selection in the context of scRNA-seq data, including the process of extracting importance scores. (b) Some notations used in the description of the deep learning methods are unclear and should be clearly defined for better understanding.

Response: We thank the suggestion from the reviewer. We have now included more details on the set up of deep learning-based feature selection methods in the revised manuscript. This includes a new section on “Setup of deep learning-based feature selection workflow”. We have also improved on the notation used in describing the deep learning methods throughout the manuscript (Section “Deep learning-based feature selection methods”). We hope these revisions provide more clarity and enhance the readability of the manuscript.

4. In the section on "Feature selection reproducibility within and across feature selection methods," the authors should provide further justification for why having a diversified expression in the selected genes is desirable. Additional explanation will help readers understand the rationale behind this criterion.

Response: As suggested, we have now discussed the rationale in the revised manuscript (first paragraph, section “Discussion and conclusion”). In essence, deep learning-based methods implemented in this work are classified as embedded feature selection methods while differential distribution-based methods are classified as univariate filter methods (PMID: 34847932). It is known that embedded feature selection methods evaluate groups of features simultaneously and therefore can capture genes that are diverse in their expressions and together may complement each other for cell type classification. In comparison, univariate filter feature selection methods evaluate each gene separately and can lead to selecting genes with similar profiles and therefore may be redundant in terms of the information they provide to the classification model.

5. The manuscript does not include some popular statistical methods for scRNA-seq analysis, such as the Wilcoxon test used in the widely-used scRNA-seq package Seurat. Considering the popularity of these methods, it would be valuable to include them in the comparison to provide a comprehensive evaluation.

Response: We thank the reviewer for this suggestion. We have now included the Wilcoxon rank-sum test in our evaluation. We have also included other popular methods such as the RandomForest and RelieF algorithms for feature selection in our experiments and have updated all figures and the manuscript to incorporate these new results.

**Second round of review**

**Reviewer 3**

The authors have addressed my comments satisfactorily.
